# Mapping RANKL- and OPG-expressing cells in bone tissue: the bone surface cells as activators of osteoclastogenesis and promoters of the denosumab rebound effect

**DOI:** 10.1038/s41413-024-00362-4

**Published:** 2024-10-18

**Authors:** Bilal M. El-Masri, Christina M. Andreasen, Kaja S. Laursen, Viktoria B. Kofod, Xenia G. Dahl, Malene H. Nielsen, Jesper S. Thomsen, Annemarie Brüel, Mads S. Sørensen, Lars J. Hansen, Albert S. Kim, Victoria E. Taylor, Caitlyn Massarotti, Michelle M. McDonald, Xiaomeng You, Julia F. Charles, Jean-Marie Delaisse, Thomas L. Andersen

**Affiliations:** 1 Department of Clinical Research, University of Southern Denmark, Odense, Denmark; 2 Danish Spatial Imaging Consortium, University of Southern Denmark, Odense, Denmark; 3 Department of Pathology, Odense University Hospital, Odense, Denmark; 4 Department of Forensic Medicine, Aarhus University, Aarhus, Denmark; 5 Department of Biomedicine, Aarhus University, Aarhus, Denmark; 6 Department of Otorhinolaryngology – Head and Neck Surgery and Audiology, University Hospital of Copenhagen, Rigshospitalet, Copenhagen, Denmark; 7 Skeletal Diseases Program, Garvan Institute of Medical Research, Sydney, NSW Australia; 8 Cancer Theme, School of Medical Sciences, Faculty of Medicine and Health, The University of Sydney, Sydney, Australia; 9 Department of Orthopedic Surgery, Brigham and Women’s Hospital, Harvard Medical School, Boston, MA USA

**Keywords:** Osteoporosis, Bone, Osteoporosis

## Abstract

Denosumab is a monoclonal anti-RANKL antibody that inhibits bone resorption, increases bone mass, and reduces fracture risk. Denosumab discontinuation causes an extensive wave of rebound resorption, but the cellular mechanisms remain poorly characterized. We utilized in situ hybridization (ISH) as a direct approach to identify the cells that activate osteoclastogenesis through the RANKL/OPG pathway. ISH was performed across species, skeletal sites, and following recombinant OPG (OPG:Fc) and parathyroid hormone 1–34 (PTH) treatment of mice. OPG:Fc treatment in mice induced an increased expression of RANKL mRNA mainly in trabecular, but not endocortical bone surface cells. Additionally, a decreased expression of OPG mRNA was detected in bone surface cells and osteocytes of both compartments. A similar but more pronounced effect on RANKL and OPG expression was seen one hour after PTH treatment. These findings suggest that bone surface cells and osteocytes conjointly regulate the activation of osteoclastogenesis, and that OPG:Fc treatment induces a local accumulation of osteoclastogenic activation sites, ready to recruit and activate osteoclasts upon treatment discontinuation. Analysis of publicly available single-cell RNA sequencing (scRNAseq) data from murine bone marrow stromal cells revealed that *Tnfsf11*^+^ cells expressed high levels of *Mmp13*, *Limch1*, and *Wif1*, confirming their osteoprogenitor status. ISH confirmed co-expression of *Mmp13* and *Tnfsf11* in bone surface cells of both vehicle- and OPG:Fc-treated mice. Under physiological conditions of human/mouse bone, RANKL is expressed mainly by osteoprogenitors proximate to the osteoclasts, while OPG is expressed mainly by osteocytes and bone-forming osteoblasts.

## Introduction

Bone remodeling consists of a highly coordinated sequence of events including (i) activation, (ii) initial bone resorption by primary osteoclasts, (iii) reversal-resorption where secondary osteoclasts intermixed with osteoblastic reversal cells extend the bone resorption phase and prepare the bone surfaces for (iv) bone formation by osteoblasts.^1–3^ An uncoupling of this process, where bone resorption occurs with a delayed subsequent bone formation, causes a net bone loss, ultimately leading to bone diseases like osteoporosis.^4,5^ Today, anti-resorptive drugs like denosumab have become first-line of treatment for osteoporosis.^6,7^ Denosumab, a monoclonal antibody against RANKL, efficiently inhibits osteoclast development and resorption, increases bone mineral density (BMD), and reduces fracture risk in patients with postmenopausal osteoporosis.^8–10^ Paradoxically, denosumab discontinuation has been associated with extensive bone resorption, leading to a drop in BMD, returning to or even below baseline levels.^8,11^ This extensive bone resorption upon treatment discontinuation, commonly referred to as “rebound resorption”, increases the risk of multiple vertebral fractures.^12–16^

The mechanism leading to rebound resorption remains largely unresolved. Multiple studies have emerged, suggesting possible mechanisms responsible for this phenomenon. In humans, the suggested mechanisms include an increased pool of circulating pre-osteoclasts,^17^ and an increased proportion of empty osteocyte lacunae, reflecting an increased pool of dead osteocytes during denosumab treatment.^18^ In murine models, it has been shown that recombinant OPG:Fc treatment, like denosumab, inhibits osteoclast development resulting in fission of osteoclasts into osteomorphs.^19^ Osteomorphs may accumulate during treatment and re-fuse into functional osteoclasts upon treatment discontinuation, leading to extensive bone resorption.^19^ Recently, it has been suggested that long term denosumab treatment of mice expressing human RANKL leads to loss of the newly embedded osteocytes and their expression of OPG, possibly setting the stage for rebound bone loss upon treatment discontinuation.^20^ However, further studies are needed to directly pinpoint the cells that orchestrate the activation of osteoclastogenesis, both under physiological conditions and before the rebound resorption following denosumab treatment.

In order to gain a deeper insight into the cellular mechanisms that orchestrate the activation of osteoclastogenesis, and the subsequent rebound resorption observed after denosumab discontinuation, it is critical to identify the specific cell populations that provide the necessary signaling either stimulating or inhibiting osteoclastogenesis – and to understand their spatial organization within the bone microenvironment. Previous studies, with some dating back to the late 1990’s and early 2000’s have localized *Tnfsf11* mRNA expression to chondrocytes, osteoblasts and bone lining cells in mice,^21,22^ while *Tnfsf11* and *Tnfrsf11b* mRNA have been localized to chondrocytes, osteoblasts, lining cells, and early osteocytes in rats.^23^ In vitro studies exploring variations in RANKL and OPG expression during osteoblast differentiation suggest that RANKL is mostly expressed in early osteoblasts, while OPG is mostly expressed in mature osteoblasts.^24,25^ More recent functional studies using established *Dmp1-cre* and *Sost*-cre knockout models suggest that osteocytes are the main source of RANKL, while osteoblasts are the main source of OPG.^26,27^ However, uncertainty arises, as *Dmp1* and *Sost* promoters are not exclusively activated in one specific cell population.^20,22,27–29^ Therefore, the precise spatial distribution of the most abundant sources of RANKL and OPG, and how they are affected during RANKL-inhibiting treatment remain largely unresolved. The present study aimed to investigate the hypothesis that osteoclastogenesis is regulated conjointly by bone surface osteoprogenitors and osteocytes through local regulation of the RANKL/OPG-pathway. Moreover, we hypothesized that accumulation of local osteoclastogenic activation sites, here defined as upregulated *Tnfsf11* and/or downregulated *Tnfrsf11b* mRNA expression in osteoblastic cells localized near the bone surfaces, is a key driver of rebound resorption upon discontinuation of denosumab treatment. To address this, we utilized highly sensitive in situ hybridization (ISH) as a direct approach to spatially identify the cells that orchestrate the activation of osteoclastogenesis. This was performed by investigating the cellular expression of the murine *Tnfsf11* and human *TNFSF11* gene, encoding RANKL and the murine *Tnfrsf11b and* human *TNFRSF11B* gene, encoding OPG, as well as their prevalence after two weeks of OPG:Fc treatment. Moreover, previous studies have shown that acute PTH treatment is capable of rapidly increasing *Tnfsf11* and decreasing *Tnfrsf11b* expression in osteoblastic cells in vitro.^30,31^ Based on these findings, we utilized a single injection of PTH as a biological probe to address which cells respond to systemic PTH stimuli by regulating their expression of *Tnfsf11* and *Tnfrsf11b* in mice.

In brief, the cellular expression of RANKL and OPG was investigated in (1) tibiae from OPG:Fc-treated C57BL/6 J female mice, (2) publicly available scRNAseq data from enriched bone marrow stroma of C57BL/6 J male mice, (3) femora from C57BL/6 J female mice treated with a single injection of PTH (1–34), (4) vertebrae from male and female C57BL/6 J mice, (5) temporal bones of Sprague-Dawley rats, (6) human cortical bone specimens obtained from the proximal femur of healthy adolescents, and (7) human iliac crest bone biopsies from healthy adults.

## Results

### OPG:Fc treatment potently increases BMD and ablates osteoclasts from bone surfaces in mice, while treatment withdrawal leads to rebound resorption

**Fig. 1:**
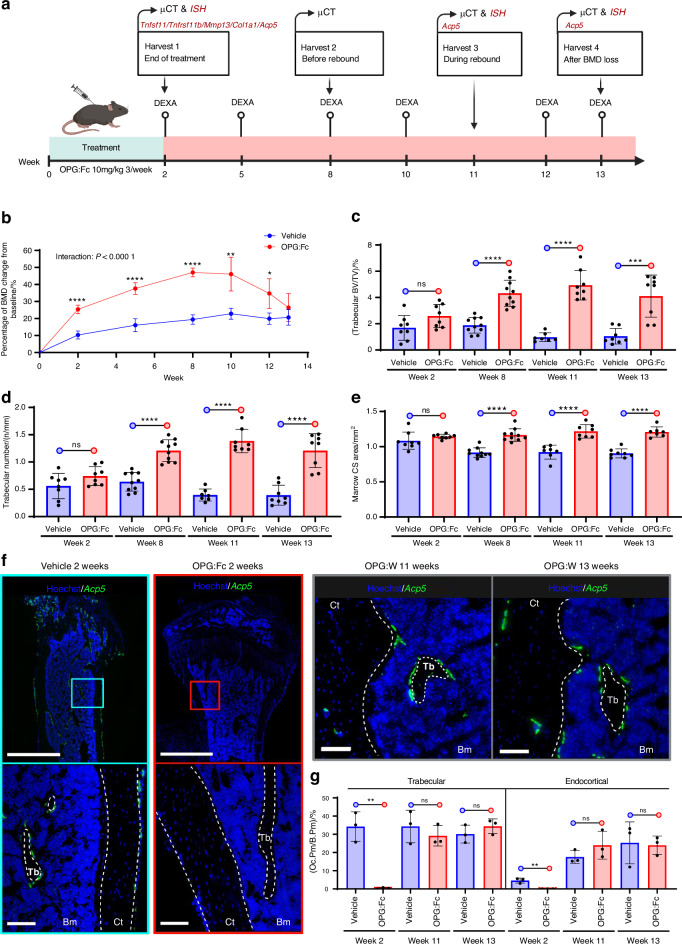
OPG:Fc treatment Increases BMD and ablates osteoclasts, while treatment withdrawal results in rebound resorption. **a** Study design illustrating 6–8-week-old, female mice that were injected with vehicle or OPG:Fc (10 mg/kg) 3 times a week for 2 weeks followed by a withdrawal period. **b** Longitudinal DEXA scans of the left hind limb were carried out every second or third week, and at the end of the study. **c**–**e** Ex vivo µCT femoral parameters at week 2, 8 11 or 13. **f** ISH of *Acp5* using tissue sections of the tibia shows *Acp5*^+^ osteoclasts (green) present on trabecular and endocortical bone surfaces in vehicle-treated but not in OPG:Fc-treated mice, with return of *Acp5*^+^ cells following treatment withdrawal. **g** Oc.Pm/B.Pm determined using *Acp5* ISH-stained sections after 2 weeks of treatment and at week 11 and 13 following withdrawal. **f** White dashed lines highlight trabecular, endocortical, and periosteal bone surfaces. **c**–**g** Data is expressed as mean ± SD. *P* values are calculated using a mixed-effects analysis in (**b**), and using an unpaired *t* test in (**c**–**e**), and (**g**). ****P values < 0.000 1, ***P values < 0.001, **P values < 0.01, *P values < 0.05. *n* = 3–8/group. Scale bars: (**f**) overview = 1 mm and high magnifications = 100 µm. Ct cortical, Tb trabecular, Bm bone marrow, OPG:W OPG:Fc withdrawal, Oc.Pm/B.Pm osteoclast perimeter/Bone perimeter, CS cross-sectional

To identify the cellular mechanisms that activate osteoclastogenesis and to address whether blocking RANKL may induce an increased abundance of local activation sites, we injected healthy 6–8-week-old female mice with OPG:Fc or vehicle 3 times a week for 2 weeks followed by treatment withdrawal, as illustrated in the study design (Fig. 1a). Longitudinal dual-energy X-ray absorptiometry (DEXA) revealed a significant interaction between time and treatment, with an initial increase in BMD, peaking at week 8 due to a prolonged half-life of OPG:Fc, followed by a decrease in BMD to vehicle levels by week 13 (Fig. 1b). Tibiae and femora were harvested following the 2 weeks of treatment, and at 8, 11, and 13 weeks after baseline (6, 9, and 11 weeks after withdrawal, respectively) (Fig. 1a). Micro-computed tomography (µCT) analysis of the femora revealed a notable increase in trabecular bone volume and trabecular thickness, accompanied by a decrease in trabecular separation at week 8, 11, and 13, in OPG:Fc-treated mice compared to vehicle, while trabecular thickness was increased at all time points compared to vehicle (Fig. 1c, d and Fig. S1a, b). Of note, at week 13, the distinction in trabecular architecture was less pronounced, suggesting a relative decrease in trabecular volume during the rebound bone loss. µCT-analysis further showed an increase in the diaphyseal marrow cross-sectional areas at week 8, 11, and 13, while total diaphyseal cross-sectional areas were increased at all timepoints compared to vehicle. This suggests that OPG:Fc treatment induced periosteal apposition and increased endosteal resorption during withdrawal (Fig. 1e and Fig. S1c). To confirm the absence of osteoclasts after OPG:Fc treatment and their reoccurrence after treatment withdrawal, we examined the presence of *Acp5*^*+*^ (encodes the osteoclast marker enzyme tartrate-resistant acid phosphatase [TRAcP]) osteoclasts on trabecular and endocortical bone surfaces using ISH. After 2 weeks, OPG:Fc treatment, like denosumab, successfully ablated *Acp5*^+^ osteoclasts from bone surfaces, while treatment withdrawal resulted in recruitment of *Acp5*^+^ osteoclasts to the bone surfaces at 11 and 13 weeks. Importantly, histological assessment revealed that *Acp5*^+^ osteoclasts predominantly occupied trabecular bone surfaces at 13 weeks during the rebound period, while endocortical bone surfaces were occupied by less osteoclasts in the OPG:Fc-treated mice (Fig. 1f, g and Fig. S2).

### *Tnfsf11* and *Tnfrsf11b* are expressed by distinct cell populations in mice

**Fig. 2:**
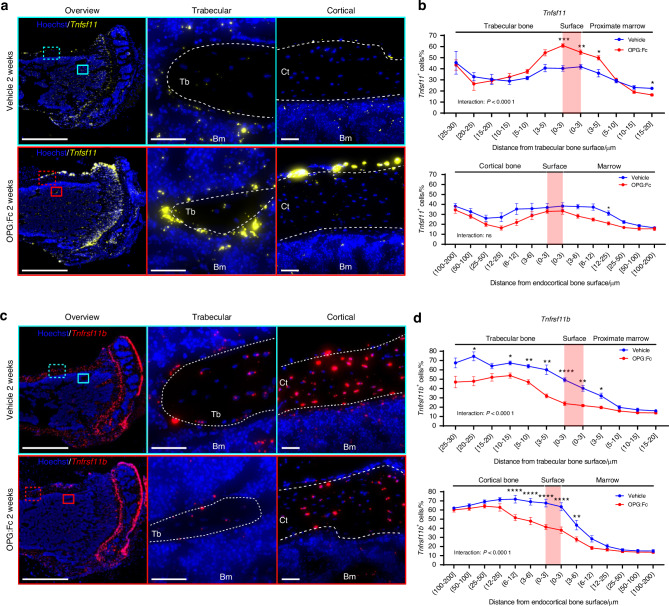
OPG:Fc treatment induces a changed *Tnfsf11* and *Tnfrsf11b* expression pattern. Tibiae from 8-week-old female mice were harvested after being injected thrice weekly for two weeks with either vehicle or OPG:Fc (10 mg/kg). Following the final dose of OPG:Fc at the end of the two-week period, *Tnfsf11* and *Tnfrsf11b* ISH was performed on tissue sections. **a** *Tnfsf11* (yellow) illustrated in bone sections from vehicle and OPG:Fc-treated mice. **b** Percentage of *Tnfsf11*^+^ cells against the distance from the trabecular or endocortical bone surface. **c** *Tnfrsf11b* (red) illustrated in bone sections from vehicle and OPG:Fc-treated mice. **d** Percentage of *Tnfrsf11b*^+^ cells against the distance from the trabecular or endocortical bone surface. **a**, **c** White dashed lines highlight trabecular, endocortical, and periosteal bone surfaces. Data is expressed as mean ± SEM. *P* values are calculated using a two-way ANOVA. ****P values < 0.000 1, ***P values < 0.001, **P values < 0.01, *P values < 0.05. *n* = 8/group. Scale bars: (**a**, **b**) overview = 1 mm and high magnifications (**a**) = 100 µm, (**b**) = 50 µm. Ct cortical, Tb trabecular, Bm bone marrow, OPG:W OPG:Fc withdrawal

Next, we investigated the expression of *Tnfsf11* and *Tnfrsf11b* to localize the cells that activate osteoclastogenesis. We found that *Tnfsf11* and *Tnfrsf11b* expression is differently distributed when considering the zones beneath, above, and at the bone surfaces. The expression of *Tnfsf11* was most prominent in bone surface cells located on periosteal, endocortical, and trabecular bone surfaces, which to our knowledge has not previously been directly illustrated (Fig. 2a). To determine the location of *Tnfsf11*^+^ cells we quantified the percentage of *Tnfsf11*^+^ cells and measured the distance of these cells to trabecular and endocortical bone surfaces. In vehicle-treated mice, we found that the percentage and staining intensities of *Tnfsf11*^+^ cells remained relatively stable across different distances from the bone surfaces, with a slight increase near the bone surface and deeper osteocytes (Fig. 2b and Fig. S3a). Likewise, we examined the spatial expression of *Tnfrsf11b. Tnfrsf11b* ISH revealed intense expression mainly amongst osteocytes, and to a lesser extent in the bone surface cells. Moreover, *Tnfrsf11b*^+^ cell populations residing in the marrow seemed less abundant (Fig. 2c). We further quantified the percentage of *Tnfrsf11b*^*+*^ cells and measured the distance of these cells from trabecular and endocortical bone surfaces. In vehicle-treated mice, osteocytes and cells near the bone surface were the most abundant population of *Tnfrsf11b*^*+*^ cells in both cortical and trabecular bone, while a lower proportion of *Tnfrsf11b*^*+*^ cells resided in the marrow (Fig. 2d). A similar pattern was seen in *Tnfrsf11b* staining intensities (Fig. S3b).

### OPG:Fc treatment induces a change in the *Tnfsf11* and *Tnfrsf11b* expression pattern in mice

Since denosumab treatment is associated with rebound bone loss upon treatment discontinuation, we hypothesized that denosumab treatment may alter the expression of *Tnfsf11* and *Tnfrsf11b*. Therefore, we examined the expression of *Tnfrsf11* and *Tnfrsf11b* at the end of 2 weeks OPG:Fc treatment. ISH revealed a noticeable increase in the expression of *Tnfsf11* especially on trabecular bone surfaces and the periosteum of the cortex, while endocortical surfaces seemed less affected (Fig. 2a). We further quantified the percentage of *Tnfsf11*^*+*^ cells and the *Tnfsf11* staining intensity and found a significant interaction between regions and treatment (Fig. 2b and Fig. S3a). Moreover, the percentage of *Tnfsf11*^*+*^ cells and the *Tnfsf11* staining intensity increased significantly on the trabecular bone surface in OPG:Fc-treated mice compared to vehicle-treated mice (Fig. 2b and Fig. S3a), suggesting that *Tnfsf11* expression is mainly upregulated in trabecular bone surface cells upon OPG:Fc treatment. Examining the abundance of *Tnfsf11* expressing cells in the cortical compartment, no interaction between region and treatment was detected. Moreover, the abundance of *Tnfsf11*^+^ cells was not increased in OPG:Fc-treated mice (Fig. 2b). However, interaction between treatment and region was detected when comparing staining intensities between vehicle- and OPG:Fc-treated mice across the regions (Fig. S3a). In contrast to the expression of *Tnfsf11*, the expression of *Tnfrsf11b* was strongly reduced upon OPG:Fc treatment in both the cortical and trabecular compartment (Fig. 2c). We further quantified the percentage of *Tnfrsf11b*^+^ cells and the *Tnrfsf11b* staining intensity and found a significant interaction between regions and treatment (Fig. 2d and Fig. S3b). Finally, the abundance of *Tnfrsf11b*^+^ cells was persistently lower on the trabecular and endocortical bone surfaces and among osteocytes residing close to the surface in OPG:Fc-treated mice compared to vehicle (Fig. 2d), while *Tnrfsf11b* staining intensity was lower in almost all cortical and trabecular osteocytes in OPG:Fc-treated mice compared to vehicle (Fig. S3b).

### OPG:Fc treatment in mice induces an increase in *Tnfsf11* and a decrease in *Tnfrsf11b* expression in the trabecular bone

Since denosumab discontinuation is associated with multiple spontaneous vertebral fractures, we hypothesized that the rebound mainly affects trabecular and not cortical bone.^9^ Therefore, we aimed to assess the expression of *Tnfsf11* and *Tnfrsf11b* across cell types and in the two bone compartments. In each compartment, we defined three distinct cell types based on their spatial position. Cells residing on the bone surface, including bone lining cells, osteoblasts, and reversal cells were collectively defined as bone surface cells. Moreover, bone matrix embedded cells were defined as osteocytes, while cells residing in the marrow were defined as marrow cells. Then, we examined the impact of 2 weeks of OPG:Fc treatment on these cell types.

**Fig. 3:**
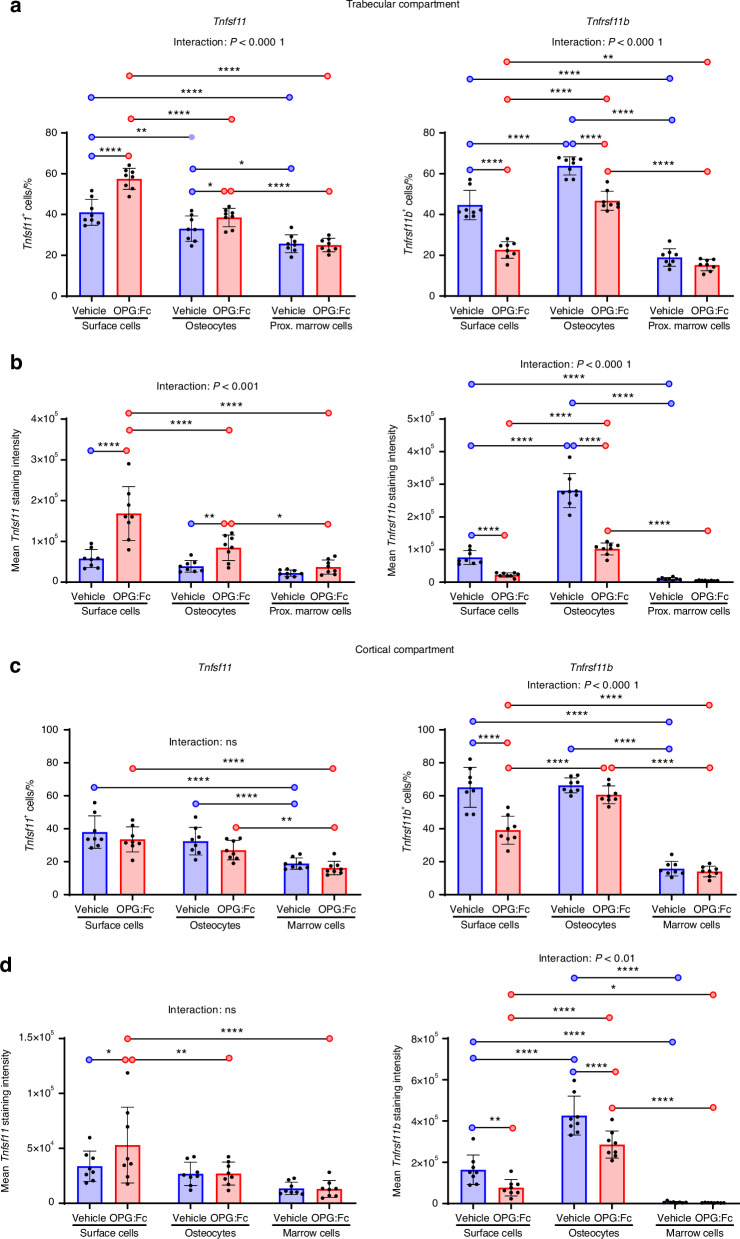
OPG:Fc treatment has differential effects on *Tnfsf11* and *Tnfrsf11b* expression in cortical and trabecular bone. *Tnfsf11*^*+*^ and *Tnfrsf11b*^+^ cell percentage and cell staining intensities were quantified in bone surface cells, osteocytes, and marrow cells and compared across cell types and between vehicle and OPG:Fc-treated mice. All data is collected from ISH stained sections of the tibia. **a** Percentage of *Tnfsf11*^***+***^ and *Tnfrsf11b*^+^ trabecular surface cells, trabecular osteocytes, and proximate marrow cells. **b** Mean *Tnfsf11* and *Tnfrsf11b* cell staining intensities in trabecular surface cells, trabecular osteocytes and proximate marrow cells **c** Percentage of *Tnfsf11*^***+***^ and *Tnfrsf11b*^+^ endocortical surface cells, cortical osteocytes and marrow cells. **d** Mean *Tnfsf11* and *Tnfrsf11b* cell staining intensities in endocortical surface cells, cortical osteocytes, and marrow cells. Data are shown as mean ± SD. *P* values are calculated using a two-way ANOVA. ****P values < 0.000 1, ***P values < 0.001, **P values < 0.01, *P values < 0.05. *n* = 8/group

In the trabecular compartment, we found that the vehicle-treated mice had a significantly higher proportion of trabecular bone surface cells expressing *Tnfsf11*^*+*^ compared to osteocytes and proximate marrow cells. Next, we compared the percentage of *Tnfsf11*^*+*^ in vehicle and OPG:Fc-treated mice and found that *Tnfsf11*^*+*^ was strongly upregulated among bone surface cells compared to vehicle. Notably, the percentage of *Tnfsf11*^*+*^ bone surface cells were significantly higher compared to osteocytes, and proximate marrow cells in OPG:Fc treated mice (Fig. 3a). Although less prominent, the percentage of *Tnfsf11*^*+*^ osteocytes were also significantly increased in OPG:Fc-treated mice compared to vehicle-treated mice (Fig. 3a). No difference was detected in proximate marrow cells between vehicle and OPG:Fc treated mice (Fig. 3a). We further quantified the percentage of *Tnfrsf11b*^*+*^ cells and found a higher proportion of osteocytes expressing *Tnfrsf11b*^*+*^ compared to bone surface cells and proximate marrow cells in vehicle-treated mice. In addition, surface cells also presented with a higher percentage of *Tnfrsf11b*^*+*^ compared to proximate marrow cells (Fig. 3a). Similarly, we examined the effect of OPG:Fc treatment on *Tnfrsf11b* expression. Here, we found that OPG:Fc treatment resulted in a significant decrease in the percentage of *Tnfrsf11b*^*+*^ bone surface cells and osteocytes (Fig. 3a). No difference was detected between proximate marrow cells upon OPG:Fc treatment (Fig. 3a). The increase in *Tnfsf11*^*+*^ and decrease in *Tnfrsf11b*^*+*^ cells resulted in a significantly increased *Tnfsf11*^*+*^*/Tnfrsf11b*^*+*^ cell ratio among all cell types upon OPG:Fc treatment, with the most prominent increase observed in bone surface cells (Fig. S3c). Examining the changes in *Tnfsf11* and *Tnfrsf11b* on histological sections, the *Tnfsf11 and Tnfrsf11b* staining intensity appeared highly affected by OPG:Fc treatment (Fig. 2a, c). Therefore, we quantified the mean staining intensity and found that the *Tnfsf11* staining intensity was increased almost 3-fold in bone surface cells, and by 2-fold in osteocytes upon OPG:Fc treatment (Fig. 3b). Notably, bone surface cells exhibited a significantly higher staining intensity compared to osteocytes and proximate marrow cells in the OPG:Fc-treated mice (Fig. 3b). *Tnfrsf11b* staining intensity was decreased almost 3-fold in bone surface cells, more than 3-fold in osteocytes, and were unaltered in marrow cells (Fig. 3b). The changes in *Tnfsf11 and Tnfrsf11b* staining intensities, resulted in an 11-fold increase in the *Tnfsf11/Tnfrsf11b* staining intensity ratios amongst bone surface cells and approximately a 3-fold increase in the *Tnfsf11/Tnfrsf11b* staining intensity ratios in marrow cells proximate to the bone surface (Fig. S3c).

### OPG:Fc treatment does not result in an increased expression of *Tnfsf11* in the cortical compartment

In the cortical compartment, the percentage of *Tnfsf11*^*+*^ cells did not differ between the vehicle and the OPG:Fc-treated mice. However, a significantly higher proportion of endocortical surface cells and cortical osteocytes were *Tnfsf11*^*+*^ compared to marrow cells. Interestingly, we found a 40% reduction in *Tnfrsf11b*^*+*^ bone surface cells, while the abundance of osteocytes and marrow cells were unaffected by OPG:Fc treatment (Fig. 3c). Accordingly, *Tnfsf11*^*+*^*/Tnfrsf11b*^*+*^ cell ratios were significantly higher among bone surface cells in the OPG:Fc-treated mice compared to vehicle, but were unchanged in osteocytes and marrow cells (Fig. S3d). Like in the trabecular bone, we examined the effect of OPG:Fc treatment on *Tnfsf11* and *Tnfrsf11b* staining intensity on cortical osteocytes, endocortical surface cells, and marrow cells (Fig. 3d). In the OPG:Fc-treated mice, *Tnfsf11* staining intensity in bone surface cells was increased by approximately 1.5-fold compared to vehicle. Moreover, *Tnfsf11* staining intensity was higher in bone surface cells than in osteocytes and marrow cells, in the OPG:Fc-treated mice (Fig. 3d). *Tnfrsf11b* staining intensity was higher in cortical osteocytes than in endocortical surface cells and marrow cells, and was significantly reduced in cortical osteocytes and endocortical surface cells upon OPG:Fc treatment (Fig. 3d). *Tnfsf11/Tnfrsf11b* staining intensity ratios seemed unaffected by OPG:Fc treatment, but were generally higher in marrow cells (Fig. S3d).

### *Tnfrsf11b* expression by chondrocytes in the growth plate and articular cartilage is high compared to the primary spongiosa, where it becomes even lower upon OPG:Fc treatment

**Fig. 4:**
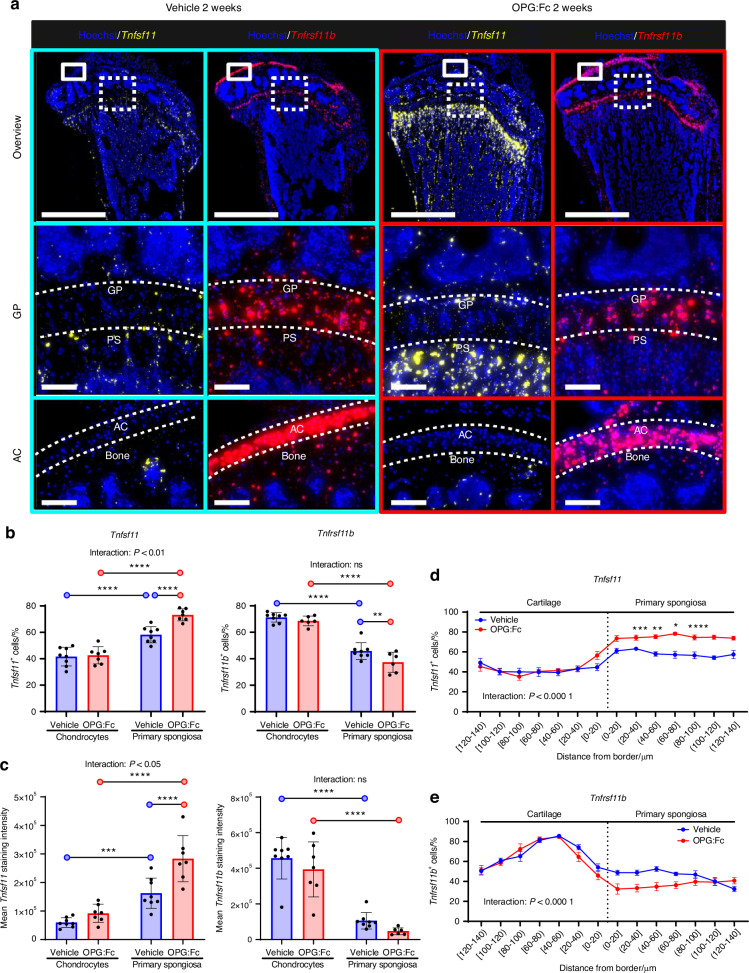
Chondrocytes of the epiphysial growth plate of the tibia express high levels of *Tnfrsf11b*, while cells residing in the primary spongiosa express high levels of *Tnfsf11*. All results on this figure are female mice treated with vehicle or OPG:Fc for two weeks. **a** ISH of *Tnfrsf11b* (red) present at high levels in the growth plate of the tibia and articular cartilage and *Tnfsf11* (yellow), present at the primary spongiosa. **b** Percentage of *Tnfsf11*^*+*^ and *Tnfrsf11b*^+^ cells, and *Tnfsf11*^+^*/Tnfrsf11b*^+^ cell ratios in chondrocytes and cells within the primary spongiosa. **c** Mean staining intensities of *Tnfsf11* and *Tnfrsf11b*, and *Tnfsf11/Tnfrsf11b* staining intensity ratios in chondrocytes and cells within the primary spongiosa. **d**, **e** Percentage of *Tnfsf11*^+^ and *Tnfrsf11b*^+^ cells against the distance from the border between the primary spongiosa and chondrocytes. **b**, **c** Data are shown as mean ± SD. **d**, **e** Data are shown as mean ± SEM. *P* values are calculated using a two-way ANOVA or a mixed-effects analysis. **b**–**e** ****P values < 0.000 1, ***P values < 0.001, **P values < 0.01, *P values < 0.05. *n* = 8/group. Scale bars: Overview = 1 mm, high magnification images = 100 µm. GP growth plate, AC articular cartilage, PS primary spongiosa

We explored other local sources of RANKL and OPG. ISH for *Tnfsf11* and *Tnfrsf11b* revealed a prominent expression of *Tnfrsf11b* in chondrocytes of the epiphyseal growth plate and the articular cartilage, while *Tnfsf11* expression seemed higher in the primary spongiosa below the growth plate (Fig. 4a). Moreover, *Tnfsf11* expression seemed to be upregulated upon OPG:Fc treatment in the primary spongiosa (Fig. 4a). Quantification of the *Tnfsf11* and *Tnfrsf11b expression* in the chondrocytes of the growth plate and primary spongiosa revealed that approximately 40% of chondrocytes expressed *Tnfsf11*, while the proportion of *Tnfsf11*^*+*^ cells increased to almost 60% in the primary spongiosa immediately below the growth plate (Fig. 4b). The proportion of *Tnfsf11*^*+*^ cells increased in the primary spongiosa, but not in chondrocytes, upon OPG:Fc treatment. In contrast, the proportion of *Tnfrsf11b*^*+*^ cells were higher in chondrocytes than in the primary spongiosa (Fig. 4b). OPG:Fc treatment significantly reduced the proportion of *Tnfrsf11b*^*+*^ cells in the primary spongiosa (Fig. 4b). Moreover, OPG:Fc treatment increased the *Tnfsf11*^*+*^*/Tnfrsf11b*^*+*^ cell ratio significantly in the cells in the primary spongiosa, but not in the chondrocytes (Fig. S4a). To further examine the expression of *Tnfsf11* and *Tnfrsf11b*, we quantified staining intensities and found a higher *Tnfsf11* staining intensity in the primary spongiosa than in chondrocytes, while *Tnfrsf11b* staining intensity was higher in chondrocytes (Fig. 4c). Moreover, *Tnfsf11* staining intensity was enhanced by OPG:Fc treatment, while *Tnfrsf11b* staining intensities were generally not affected (Fig. 4c). The *Tnfsf11/Tnfrsf11b* staining intensity ratio increased more than 4-fold in the primary spongiosa upon OPG:Fc treatment (Fig. S4a). To visualize the expression pattern of *Tnfsf11* and *Tnfrsf11b*, we quantified the percentage of positive cells, and related their distance to an interface line separating the chondrocytes from the primary spongiosa (Fig. 4d, e and Fig. S4b, c). For both *Tnfsf11* and *Tnfrsf11b*, a significant interaction between regions and treatment was detected (Fig. 4d, e and Fig. S4b, c). Moreover, The percentage of *Tnfsf11*^+^ cells and *Tnfsf11* staining intensities remained low in the cartilage, and increased as the tissue transitioned to primary spongiosa, with OPG:Fc treatment increasing the *Tnfsf11*^+^ cells and *Tnfsf11* staining intensity in the primary spongiosa (Fig. 4d and Fig. S4b). Conversely, *Tnfrsf11b*^+^ cells and *Tnfrsf11b* staining intensity increased gradually in chondrocytes of the growth plate, while it decreased again in the primary spongiosa (Fig. 4e and Fig. S4c). OPG:Fc treatment induced a decrease in *Tnfrsf11b* staining intensity in the primary spongiosa (Fig. S4c).

### Bone surface osteoprogenitors expressing the gene encoding matrix metallopeptidase 13 *(Mmp13) also* express *Tnfsf11*

**Fig. 5:**
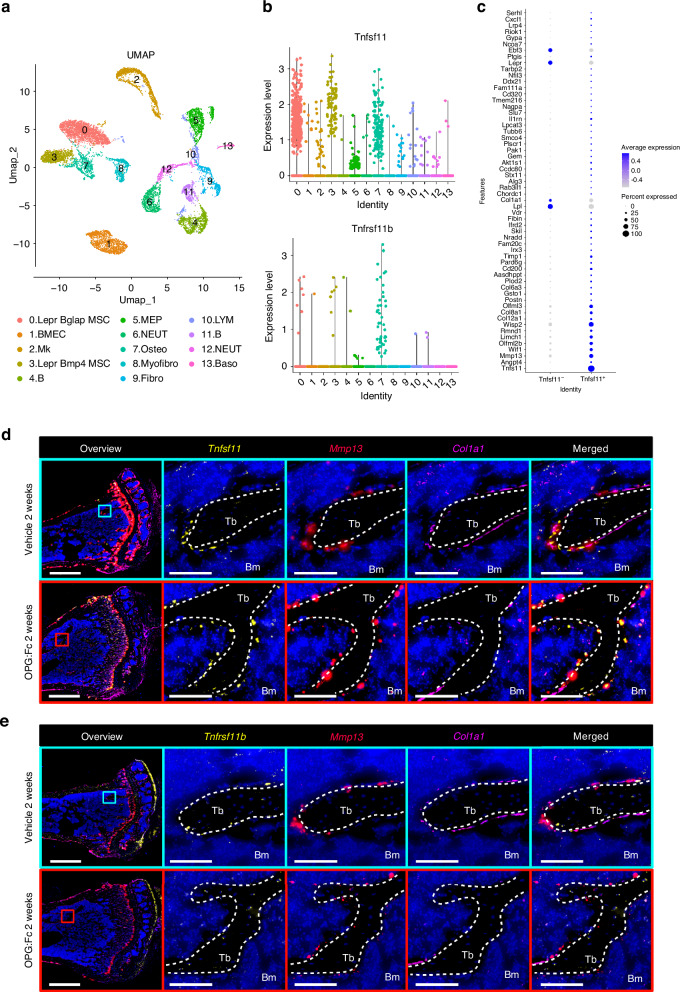
Single cell RNA-seq data of enriched bone marrow stroma of male mice and multiplex ISH of tissue sections from OPG:Fc-treated female mice. **a** Bone marrow cells were classified into 13 cell clusters by UMAP: (0) Lepr^+^ Bglap^+^ mesenchymal stem cell (MSC), (1) bone marrow endothelial cell (BMEC), (2) megakaryocyte (Mk), (3) Lepr^+^ Bmp4^hi^ MSC, (4) B cell, (5) myeloerythroid progenitor (MEP), (6) neutrophil (NEUT), (7) osteolineage cell (Osteo), (8) myofibroblast (Myofibro), (9) fibroblast (Fibro), (10) lymphocyte (LYM), (11) B cell, (12) neutrophil (NEUT), (13) basophil (Baso). **b** Expression of *Tnfrsf11b* and *Tnfsf11* within each bone marrow cell cluster. **c** All differentially expressed genes identified in *Tnfsf11*^+^ cell compared to *Tnfsf11*^−^ cells within clusters 0, 3, 7. **d** *Tnfsf11*/*Mmp13*/*Col1a1*, or (**e**) *Tnfrsf11b*/*Mmp13*/*Col1a1* multiplex ISH conducted on adjacent sections of tibiae collected from mice treated with either vehicle or OPG:Fc for two weeks. **d**, **e** White dashed lines outline trabecular bone surfaces. Tb Trabecular, Bm bone marrow

To identify the main cell populations expressing *Tnfsf11* and *Tnfrsf11b* we utilized the previously published and publicly available scRNAseq datasets.^32^ Enriched bone marrow stromal cells were classified into 13 different cell clusters (Fig. 5a). In these 13 identified cell clusters, *Tnfsf11*^*+*^ and *Tnfrsf11b*^*+*^ cells were abundant in cluster 0, 3, and 7 (Fig. 5b). Key gene markers defining the identity of bone marrow cell clusters included *Lepr*, *Cxcl12*, *Adipoq*, and *Bglap* for cluster 0, *lepr*, *Cxcl12*, *Adipoq*, and *Bmp4* for cluster 3, and *Col1a1*, *Alpl*, *Bglap*, *Bglap2*, and *Bglap3* for cluster 7. Moreover, *Mmp13* was expressed in all three cell clusters (Fig. S5a). Since our ISH assessment showed that *Tnfrsf11b* was mainly expressed by osteocytes, and the scRNAseq data only includes bone marrow stromal cells, we decided to continue with *Tnfrsf11* for further characterization of the cell populations that express this gene. We identified 58 differentially expressed genes in *Tnfsf11*^+^ cells compared to *Tnfsf11*^−^ cells in cluster 0, 3, and 7 (Fig. 5c). Of these genes, a high proportion of *Tnfsf11*^+^ cells had a high expression of genes such as *Mmp13*, *Wisp2*, *Limch1*, and *Wif1* and a low expression of genes such as *Col1a1, Lepr*, and *Lpl* (Fig. 5c). Conversely, a high proportion of *Tnfsf11*^-^ cells had a high expression of *Col1a1, Lepr*. and *Lpl*, and a *low expression of Mmp13*, *Wisp2*, *Limch1* (Fig. 5c). Since we have previously established that osteoprogenitor cells including reversal cells, canopy cells and bone lining cells express *MMP13* in humans,^33,34^ we hypothesized that these cells also express *Tnfsf11*. Multiplex ISH of *Tnfsf11* or *Tnfrsf11b* together with *Mmp13*, and *Col1a1*, revealed that the majority of *Tnfsf11*^+^ bone surface cells were *Mmp13*^+^, and *Col1a1*^-^, and that these *Tnfsf11*/*Mmp13*^+^ cells remained on the bone surfaces after OPG:Fc treatment (Fig. 5d). *Tnfrsf11b*/*Mmp13*/*Coll1a1* multiplex ISH confirmed that *Tnfrsf11b* was mainly expressed by *Mmp13*^-^/*Col1a1*^-^ osteocytes, and showed that only a few bone surface cells were *Tnfrsf11b*^+^/*Mmp13*^+^/*Col1a1*^+^(Fig. 5e) and found some *Tnfrsf11b*^+^/ *Col1a1*^high^ osteoblasts (data not shown).

### Bone surface cells and osteocytes near the bone surface respond to systemic PTH stimulus

**Fig. 6:**
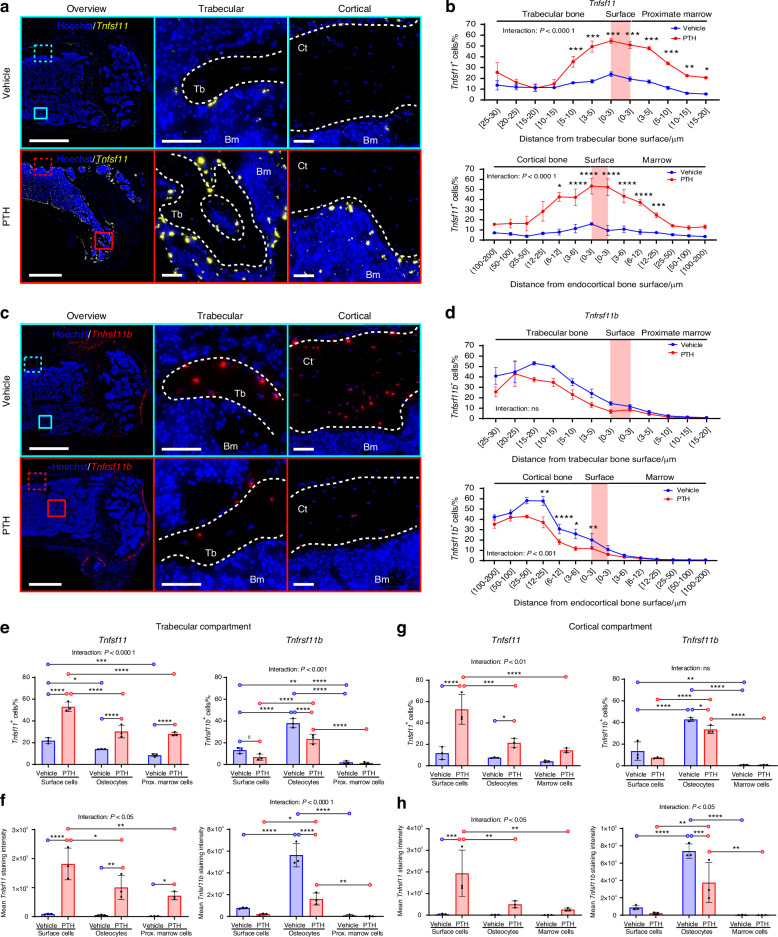
PTH treatment induces a changed *Tnfsf11* and *Tnfrsf11b* expression pattern. *Tnfsf11* and *Tnfrsf11b* ISH performed on sections of femora obtained from 12-week-old female mice, 1 h after they were injected with a single dose of PTH (80 µg/kg). **a** *Tnfsf11* (yellow) ISH illustrated in sections of the femur from vehicle and PTH treated mice. **b** Percentage of *Tnfsf11*^+^ cells against the distance from the trabecular or endocortical bone surface. **c** *Tnfrsf11b* (red) ISH illustrated in bone sections from vehicle and PTH treated mice. **d** Percentage of *Tnfrsf11b*^+^ cells against the distance from the trabecular or endocortical bone surface. **e** Percentage of *Tnfsf11*^***+***^ and *Tnfrsf11b*^+^ trabecular surface cells, trabecular osteocytes, and proximate marrow cells. **f** Mean *Tnfsf11* and *Tnfrsf11b* cell staining intensities in trabecular surface cells, trabecular osteocytes and proximate marrow cells. **g** Percentage of *Tnfsf11*^***+***^ and *Tnfrsf11b*^+^ endocortical surface cells, cortical osteocytes and marrow cells. **h** Mean *Tnfsf11* and *Tnfrsf11b* cell staining intensities in endocortical surface cells, cortical osteocytes, and marrow cells. **a**–**c** White dashed lines highlight trabecular, endocortical, and periosteal bone surfaces. In (**b**, **d**) data is expressed as mean ± SEM. In (**e**–**h**) data is expressed as mean ± SD. *P* values are calculated using a two-way ANOVA or mixed-effects analysis. ****P values < 0.000 1, ***P values < 0.001, **P values < 0.01, *P values < 0.05. *n* = 3/group. Scale bars: (**a**, **b**) Overview = 1 mm and high magnifications (**a**) = 100 µm or (**b**) = 50 µm. Ct cortical, Tb trabecular, Bm bone marrow

Previous studies suggest that *Tnfsf11* and *Tnfrsf11b* expression is regulated upon PTH treatment with the most notable effect observed one hour after treatment.^30,31^ Therefore, we utilized a single PTH treatment dose as a biological probe to induce *Tnfsf11* expression in mice, in order to (1) identify the spatial location of cells that respond to systemic PTH stimuli by regulating the expression of *Tnfsf11* and *Tnfrsf11b*, and (2) to validate that the main activator of osteoclastogenesis is cells residing near or on the bone surfaces. Histological assessment of *Tnfsf11* mRNA expression in vehicle-treated mice revealed subtle expression of *Tnfsf11* in bone surface cells, with limited expression in osteocytes. Upon PTH treatment, mRNA expression was highly increased in cells near the trabecular and endocortical bone surfaces (Fig. 6a). Next, we quantified cells and related their distances to the trabecular and cortical bone surfaces. A significant interaction between treatment and region was detected in both the trabecular and cortical compartment. Specifically, we found that PTH treatment highly increased *Tnfsf11* expression in bone surface cells, and osteocytes and marrow cells near the bone surface compared to vehicle—a finding similar to what we observed in the mice treated with OPG:Fc (Fig. 6b and Fig. S6a). Similarly, we assessed the expression of *Tnfrsf11b* using ISH and found that mainly osteocytes near the bone surfaces expressed *Tnfrsf11b* compared to marrow cells and surface cells—this osteocytic *Tnfrsf11b* expression was reduced by PTH(1–34) treatment (Fig. 6c). Quantification of *Tnfrsf11b* expressing cells, and their distances to the trabecular or endocortical bone surfaces revealed a significant interaction between treatment and region in all cases, but not when relating the abundance of *Tnfrsf11b*^+^ cells to the distance to the trabecular bone surfaces (Fig. 6d and Fig. S6b). PTH treatment reduced *Tnfrsf11b* expression in bone surface cells, and osteocytes near the bone surface compared to vehicle—this was reflected by a decreased staining intensity in trabecular osteocytes and a decrease in both the abundance and staining intensity of *Tnfrsf11b*^+^ cells in cortical osteocytes near the bone surfaces (Fig. 6d and Fig. S6b).

We subdivided the cell populations into bone surface cells, osteocytes and marrow cells to compare *Tnfsf11* and *Tnfrsf11b* expression between cell types and to determine the impact of PTH treatment on the mRNA expression. In the trabecular compartment PTH induced a 2–3-fold increase in the proportion of surface cells, osteocytes and marrow cells that expressed *Tnfsf11* (Fig. 6e). Notably, the proportion of bone surface cells expressing *Tnfsf11* was 2-fold higher compared to marrow cells and osteocytes in the PTH-treated mice (Fig. 6e). Moreover, the *Tnfsf11* staining intensities in bone surface cells, osteocytes and marrow cells were 18–35-fold higher in the PTH-treated mice compared to vehicle. Notably, bone surface cells had approximately a 2-fold higher *Tnfsf11* staining intensity than osteocytes and marrow cells after PTH-treatment (Fig. 6e). Assessing the expression of *Tnfrsf11b* across cell types in the vehicle group, we found nearly a 3-fold higher proportion of osteocytes expressing *Tnfsf11b* compared to bone surface cells, while a 20-fold higher proportion was observed in osteocytes compared to marrow cells (Fig. 6e). The proportion of osteocytes expressing *Tnfrsf11b* was significantly reduced by nearly 2-fold in osteocytes and bone surface cells after PTH treatment, while marrow cells were not affected (Fig. 6e). Similar findings in osteocytes were found for staining intensities, while the *Tnfrsf11b* staining intensities were not changed in surface cells and marrow cells in the PTH-treated mice compared to vehicle (Fig. 6f).

In the cortical compartment, a 4.5-fold higher proportion of bone surface cells expressed *Tnfsf11* in the PTH-treated compared to vehicle-treated mice (Fig. 6g). Moreover, nearly a 3-fold higher proportion of osteocytes expressed *Tnfsf11* in PTH-treated mice compared to vehicle-treated mice. No significant differences were detected in marrow cells between PTH- and vehicle-treated mice (Fig. 6g). Comparing the proportion of bone surface cells to osteocytes and marrow cells within the PTH-treated mice, the proportion of *Tnfsf11*^+^ bone surface cells was 2.5-fold higher compared to osteocytes and nearly 4-fold higher compared to marrow cells (Fig. 6g). Similar findings were found for *Tnfsf11* staining intensities (Fig. 6h). Furthermore, we assessed the expression of *Tnfrsf11b* across cell types in the vehicle group and found approximately a 3-fold higher proportion of osteocytes expressing *Tnfsf11b* compared to bone surface cells, while the proportion of *Tnfrsf11b*^+^ osteocytes was nearly 58-fold than that of marrow cells (Fig. 6g). Moreover, the proportion of osteocytes expressing *Tnfrsf11b*, and the *Tnfrsf11b* staining intensities were 1–2-fold lower in the PTH-treated mice compared to vehicle-treated mice (Fig. 6g, h).

When comparing PTH- to vehicle-treated mice, the ratio of *Tnfsf11/Tnfsf11b* positive cells and ratio of staining intensity trended toward an increase in the PTH treated group but was only significantly different for marrow cells (Fig. S6c, d).

### The inner ear of rats is protected from bone remodeling by high *Tnfrsf11b* and low *Tnfsf11* expression

**Fig. 7:**
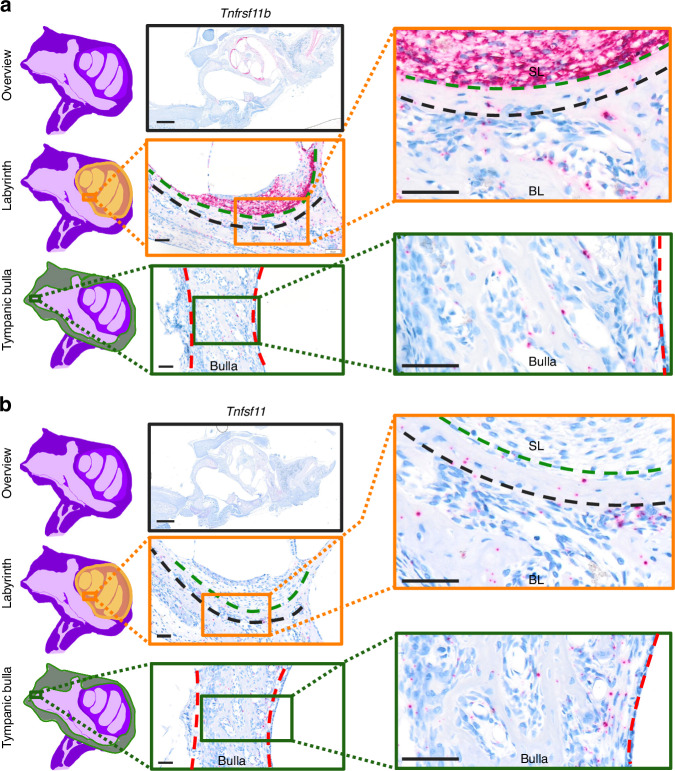
Fibrocytes of the spiral ligament express high levels of *Tnfrsf11b*. All data are obtained from a 10-day-old Sprague-Dawley rat. ISH on sections through the inner and middle ear, with representative overview image and high magnification images of the spiral ligament lining the bony labyrinth of the inner ear and the tympanic bulla of the middle ear for (**a**)*Tnfrsf11b* and (**b**) *Tnfsf11* ISH of an adjacent section. The green dashed line separates the fibrocytes of the spiral ligament from the bony labyrinth. The black dashed line separates bone protected from remodeling from bone undergoing remodeling. The red dashed line outlines a region of the tympanic bulla of the middle ear. Scale bars: overview = 1 mm, high magnification images = 50 µm. BL bony labyrinth, SL spiral ligament

To better understand the cellular mechanisms that influence the activation of osteoclastogenesis, we utilized the inner ear as a unique anatomic location. Here, bone remodeling is highly inhibited in the bone immediately surrounding the inner ear, while the bulla of the middle ear undergo bone modeling with resorption on one side and formation on the other side.^35–38^ ISH of *Tnfsf11* and *Tnfrsf11b* expression in the inner ear, revealed an extremely high local expression of *Tnfrsf11b* in the fibrocytes of the spiral ligament of the cochlea, while a minority of the osteocytes expressed *Tnfrsf11b* (Fig. 7a). In the bone immediately adjacent to the spiral ligament, bone remodeling was clearly inhibited, as bone remodeling took place at a distance from the *Tnfrsf11b*^+^ fibrocytes (Fig. 7a). We examined *Tnfsf11 expression* in adjacent to *Tnfrsf11b*-stained sections, and found little or no *Tnfsf11*^+^ cells in the spiral ligament and the bone immediately outside the spiral ligament, while *Tnfsf11*^+^ cells were increased in the bone remodeling spaces more distant from the spiral ligament, including in the bony labyrinth and in the tympanic bulla of the middle ear (Fig. 7b).

### Bone surface cells proximate to osteoclasts are the most abundant source of *Tnfsf11* in mice

**Fig. 8:**
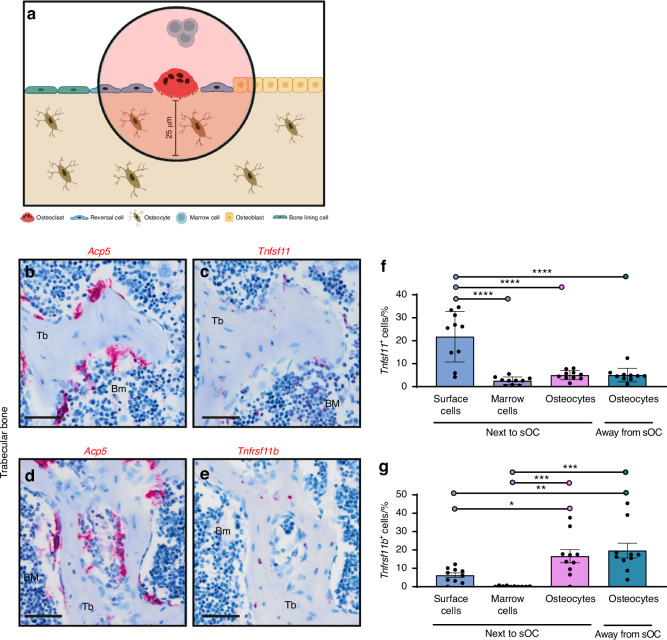
Surface cells proximate to osteoclasts mostly express *Tnfsf11* while osteocytes express *Tnfrsf11b*. Representative images in this figure are of adjacent vertebral bone sections from a 12-week-old female mouse, while quantifications are from male and female mice. **a** Methodological approach. **b** *Acp5* ISH and **c** *Tnfsf11* ISH on adjacent section. **d** *Acp5* ISH and (**e**) *Tnfrsf11b* ISH on adjacent section. Histograms illustrate the mean percentage of (**f**) *Tnfsf11*^+^ and (**g**) *Tnfrsf11b*^+^ cells. **f**, **g** Data are shown as mean ± SD and *P* values are calculated using one-way ANOVA. ****P values < 0.000 1, ***P values < 0.001, **P values < 0.01, *P values < 0.05. *n* = 10/group. Scale bars: = 50 µm. Tb Trabecular, Bm bone marrow. Illustrations were created using BioRender.com

Previously, it has been illustrated that reversal cells form cell extensions that extend beneath the bone resorbing osteoclast, demonstrating direct cell-cell contact between reversal cells and bone resorbing osteoclasts.^33,39^ Therefore, we investigated *Tnfsf11* and *Tnfrsf11b* expression in the local environment surrounding the osteoclasts in female and male mouse vertebral bone serial sections hybridized to *Tnfsf11, Tnfrsf11b, or Acp5* probes in order to characterize the environment close to the osteoclasts and to address the possibility of sexual dimorphism in *Tnfsf11* and *Tnfrsf11b* expression. We quantified the expression in cells that were up to 25 µm away from the osteoclasts residing on trabecular and endocortical bone surfaces, including mononuclear osteoblastic cells on the bone surface, marrow cells above the bone surface, and osteocytes below the bone surface. Collectively, these cells were defined as cells located next to the osteoclasts. Then, we quantified the expression of *Tnfsf11* and *Tnfrsf11b* in osteocytes located more than 25 µm away from an osteoclast (Fig. 8a). Histological images illustrate *Acp5*^+^ osteoclasts on the bone surface (Fig. 8b, d). Since we found no obvious differences in *Tnfsf11* and *Tnfrsf11b* expression between female and male mice (Fig. S7a, b), the two groups were combined to increase statistical power. *Tnfsf11*^+^ cells were mainly detected on the bone surface and only to some extent in osteocytes (Fig. 8c), while *Tnfrsf11b*^+^ cells were mainly osteocytes (Fig. 8e). Next, we quantified the proportion of *Tnfsf11*^+^ and *Tnfrsf11b*^+^ cells. A higher proportion of bone surface cells expressed *Tnfsf11*, compared to osteocytes and marrow, while no difference was detected between marrow cells and osteocytes (Fig. 8f). In contrast, the proportion of *Tnfrsf11b*^+^ cells was highest in osteocytes, but did not differ between marrow cells and surface cells. Moreover, the osteocytic expression of *Tnfrsf11b* was not affected by the proximity to osteoclasts (Fig. 8g).

### Osteoprogenitor cells proximate to the osteoclasts are the most abundant source of *TNFSF11* in humans

**Fig. 9:**
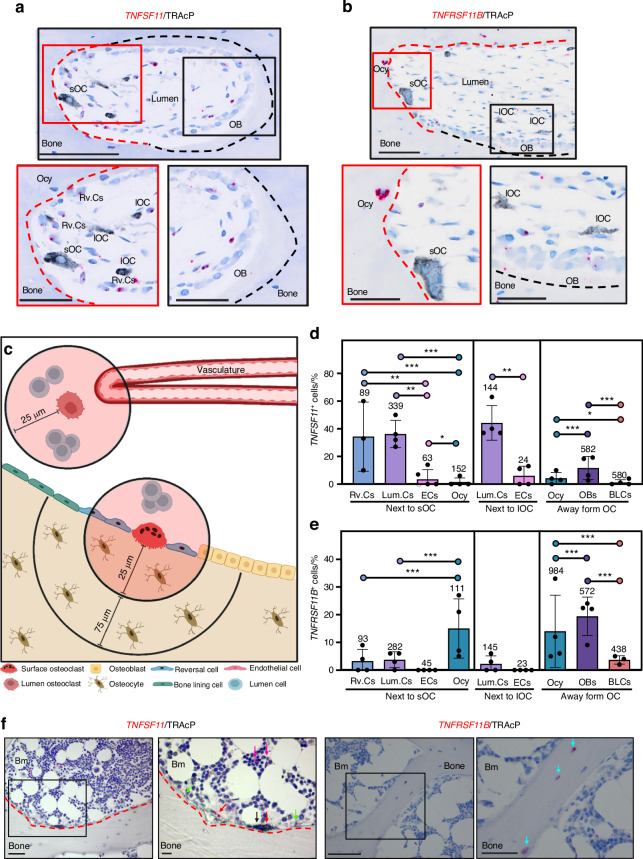
*TNFSF11* and *TNFRSF11B* expression in human cortical and trabecular bone. **a**–**e** Data is obtained from cortical femoral bone specimens acquired from 4 adolescent females, and (**f**) from iliac crest bone sections from healthy humans. **a** Representative image of a selected cutting cone from *TNFSF11* ISH (red), combined with a TRAcP (black) immunostained section. **b** Representative images of a selected cutting cone from *TNFRSF11B* (red) ISH, combined with a TRAcP (black) immunostained section. Red and black dashed lines in (**a**, **b**), respectively indicate eroded and formative surfaces, as determined by Masson’s Trichrome stained adjacent sections. **c** Methodological approach. **d** Histograms illustrate the mean percentage of *TNFSF11*^+^ cells located either next to surface osteoclasts, away from surface osteoclasts, or next to lumen osteoclasts. **e** Histogram illustrating the mean percentage of *TNFRSF11B*^+^ located either next to surface osteoclasts, away from surface osteoclasts, or next to lumen osteoclasts. The number above each bar is the number of cells quantified. **f** Representative images of human iliac crest bone sections stained for *TNFSF11* or *TNFRSF11B* ISH (red), combined with a TRAcP immunostaining (black). TRAcP^+^ Osteoclasts (black arrow), *TNFSF11*^+^ Canopy cells (red arrows), reversal cells (green arrows), marrow cells (purple arrows), and *TNFRSF11B*^+^ osteocytes (blue arrows). **d**, **e** Data are shown as mean ± SD. Clustered logistic regression was utilized to determine the likelihood of *TNFSF11* or *TNFRSF11B* being expressed in one cell population compared with another. ***P values < 0.001, **P values < 0.01, *P values < 0.05. *P* values, odds ratios, and corresponding 95% confidence intervals are shown in Table S1. Scale bars: overview = 100 µm, high magnification images = 50 µm. sOC surface osteoclast, lOC lumen osteoclast, Ocy osteocyte, OB osteoblast, Rv.C reversal cell, Lum.C lumen cell, EC vascular endothelial cell, BLC bone lining cell, Illustrations were created using BioRender.com

To determine whether a similar expression pattern exists in humans, we investigated femoral cortical bone sections from adolescents and Jamshidi iliac crest bone sections from healthy adults. Chromogenic ISH for *TNFSF11* or *TNFRSF11B* expression was combined with an immunohistochemical staining for TRAcP. Histological images illustrated the expression of *TNFSF11* in reversal cells and lumen cells residing close to osteoclasts (Fig. 9a), which previously has been demonstrated to be osteoprogenitor cells.^1,3,5,34,40^ *TNFRSF11B* was found predominantly in osteocytes close to intracortical canals and in osteoblasts on the bone surface (Fig. 9b). The cellular expression of *TNFSF11* and *TNFRSF11B* was quantified within 25 µm from surface osteoclasts or lumen osteoclasts, including mononuclear reversal cells on the bone surface, mononuclear lumen cells, and endothelial cells (Fig. 9c). Moreover, the cellular expression of *TNFSF11* and *TNFRSF11B* was quantified in osteocytes 0–100 µm away from surface osteoclasts, in osteoblasts on osteoid surfaces, and in bone lining cells on quiescent bone surfaces (Fig. 9c). Then, we calculated the likelihood of detecting *TNFSF11* and *TNFRSF11B* across different cell types. We found that reversal cells next to osteoclasts on eroded surfaces and lumen cells next to surface or lumen osteoclasts were more likely to express *TNFSF11* compared to endothelial cells of the vascular structures, osteocytes (Fig. 9d and Table S1), osteoblasts on osteoid surfaces, and bone lining cells on quiescent surfaces (Table S1). Contrary to the *TNFSF11* expression pattern, osteocytes and osteoblasts were more likely to express *TNFRSF11B* compared to reversal cells, lumen cells and bone lining cells (Fig. 9e and Table S1) Similar to the cortical compartment, histological assessment of *TNFSF11* and *TNFRSF11B* in trabecular compartment revealed that *TNFSF11* was mainly expressed by reversal cells and canopy cells close to osteoclasts, while *TNFRSF11B* was mainly expressed by trabecular osteocytes (Fig. 9f).

## Discussion

The identification of cells orchestrating the activation of osteoclastogenesis through the RANKL/OPG-pathway have been debated since the discovery of the RANKL/OPG system in the 1990s,^26,27,41–46^ but the exact and most abundant cellular sources of RANKL and OPG has remained unresolved until now. In the present study, we investigated the cellular in situ mRNA expression of *TNFSF11* (encoding RANKL) and *TNFRSF11B* (encoding OPG) across mouse, rat, and human bone, across different skeletal sites and compartments, and upon OPG:Fc treatment in mice, reflecting the events leading to rebound resorption upon denosumab discontinuation. Moreover, we utilized publicly available scRNAseq data from enriched bone marrow stoma of mice to identify and characterize cell populations that express RANKL. Finally, we utilized a single injection of PTH to map the landscape of the cells that orchestrate the activation of osteoclastogenesis through the RANKL/OPG-pathway.

**Fig. 10:**
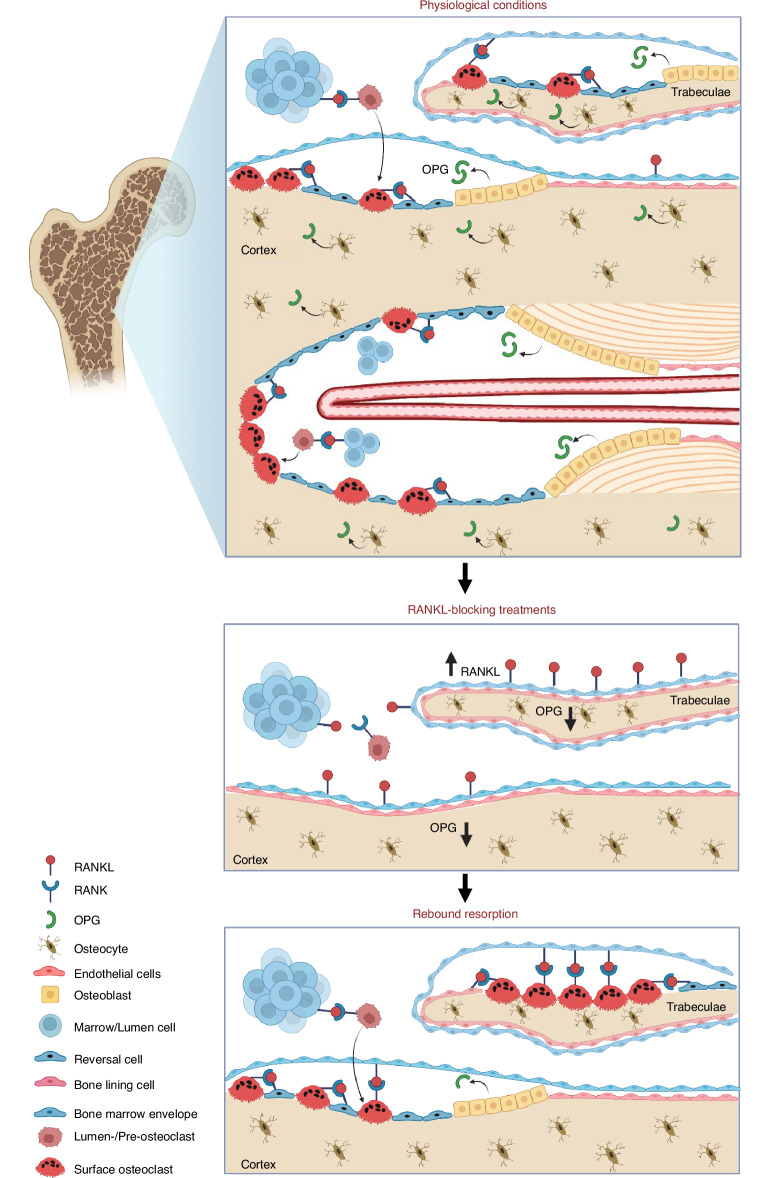
Concluding figure illustrating key findings. Under physiological conditions surfaces are activated by e.g. PTH stimulus and occupied by osteoclasts that resorb bone. Osteoprogenitors are recruited to the bone surface, where they continue providing the necessary RANKL for continuous recruitment of osteoclasts until bone formation is initiated where osteoblasts, together with osteocytes provide inhibitory OPG. During inhibition of RANKL (by e.g. OPG:Fc or denosumab treatment), osteoclasts are abolished, also leading to the abolishment of osteoblast lineage cells due to the coupling of bone resorption and formation. Moreover, trabecular bone surface cells increase the production of RANKL, while OPG production is decreased in trabecular surface cells and osteocytes. RANKL production remains unchanged in endocortical surface cells and osteocytes, while OPG is reduced in bone surface cells and osteocytes near the surface. Taken together, this reflects an accumulation of local activation sites with an increase in RANKL production in trabecular, but not endocortical bone surfaces, and a decrease in OPG in bone surface cells, and osteocytes close to the trabecular and endocortical surfaces. This shift in local RANKL/OPG production eventually sets stage for rebound bone resorption upon treatment discontinuation. Illustrations were created using BioRender.com

The study demonstrates that osteoprogenitors close to the bone surface (bone lining cells, reversal cells, and lumen/canopy cells) are the most abundant source of RANKL, whereas osteocytes, bone-forming osteoblasts, fibrocytes of the inner ear, and chondrocytes of the growth plate and articular cartilage are the most abundant source of OPG. Two weeks of OPG:Fc treatment resulted in accumulation of osteoclastic activation sites on trabecular bone surfaces, characterized by increased expression of RANKL mostly by bone surface cells and reduced local expression of OPG by bone surface cells and osteocytes. However, this was not the case on endocortical bone surfaces, where bone surface cells and osteocytes exhibited a reduced OPG expression, while RANKL expression were less affected. Accordingly, the osteoclastic rebound upon OPG:Fc withdrawal likely occurs mainly on trabecular surfaces, and not on endocortical surfaces, which probably explain why denosumab discontinuation may increase the risk of multiple vertebral fractures, as vertebrae predominately consist of trabecular bone.^47^ Taken together, these findings suggest that treatments that inhibit the function of RANKL induce an accumulation of local osteoclastogenic activation sites that are ready to induce osteoclastogenesis upon treatment withdrawal, as summarized in Fig. 10 and discussed below.

Several studies have presented novel insights into the mechanisms contributing to rebound resorption upon denosumab discontinuation, potentially leading to bone loss and multiple vertebral fractures in patients.^15,18–20,48–51^ One possible mechanism is that osteoclasts have been shown to undergo fission, forming mononucleated osteomorphs. This fission is induced by denosumab treatment, as recently shown in an OPG:Fc-treated murine model.^19^ Here, the notion is that these accumulating osteomorphs re-fuse upon treatment withdrawal, thereby contributing to the rebound resorption.^19^ In line with this, another study has reported an increased pool of circulating pre-osteoclasts during denosumab treatment.^17^ Nonetheless, the accumulation of pre-osteoclasts and osteomorphs, whether locally or in the circulation, only partially explains the rebound resorption associated with denosumab discontinuation. Pre-osteoclasts need to be recruited and stimulated to fuse and initiate bone resorption on multiple bone surfaces before excess bone resorption occurs. The present study demonstrates that OPG:Fc treatment in mice results in a local shift in the expression of RANKL/OPG mostly on the trabecular bone surfaces, resulting in extensive recruitment of osteoclasts on bone surfaces upon treatment withdrawal, in line with previous research where OPG:Fc-treated mice showed extensive resorption following treatment withdrawal.^19,52^

This spatial link between the increased RANKL/OPG expression ratio and the wave of osteoclasts strongly supports the notion that an accumulation of local activation sites, reflected by increased RANKL expression and a decrease in OPG expression near the bone surfaces, is a key driver of rebound resorption upon treatment discontinuation. The elevated RANKL/OPG ratios in serum of denosumab-treated patients suggests that a similar phenomenon probably occur in humans.^49^ The present study shows that expression of RANKL was preferentially increased in trabecular bone surface cells upon OPG:Fc, while RANKL expression in cortical bone surface cells, as well as osteocytes and marrow cells appeared less affected by the OPG:Fc treatment. Since bone resorption and formation are coupled processes,^3,49^ treatments like denosumab in humans and OPG:Fc in mice inhibit both bone resorption and formation.^19,53,54^ Therefore, the observed higher prevalence of RANKL-expressing bone surface cells in the OPG:Fc-treated mice are likely bone lining cells or reversal cells. Under normal physiological conditions, this accumulation would have resulted in recruitment and activation of an osteoclast on the bone surface, resetting these activation sites. Previous studies have emphasized the indirect, yet an important role bone lining cells play in controlling bone resorption, as they cover the quiescent bone surfaces together with the neighboring bone marrow envelope.^1,34,55^ Moreover, bone lining cells have been proposed to function as “gate-keepers” that either prevent or allow initiation of bone resorption.^56^ Firstly, bone lining cells may release collagenases such as MMP13, which digest the unmineralized lamina limitans covering the quiescent surface, exposing bone minerals to osteoclastic contacts.^57^ Secondly, bone lining cells retract from the bone surface, making the surface accessible for recruited osteoclasts to attach, fuse and resorb bone.^33,39,58–60^ Thirdly, bone lining cells re-colonize the resorbed bone surfaces as osteoblastic reversal cells (osteoprogenitors), maintaining direct contact with secondary osteoclasts deepening the erosion.^3,33,55^ Collectively, the exclusive position of bone lining cells directly on the quiescent bone surface, together with their expression of RANKL and their role in preparing the surface for osteoclastic bone resorption, supports the pivotal role of bone lining cells and reversal cells in the activation of osteoclastogenesis and rebound resorption upon denosumab discontinuation.

Under physiological conditions in mice, the RANKL-expressing cells close to osteoclasts are more likely to be bone surface cells than osteocytes or marrow cells. In humans, these RANKL-expressing cells are more likely to be osteoprogenitors, including reversal cells and lumen/canopy cells located near osteoclasts – rather than osteocytes, marrow cells, or the more distant bone-forming osteoblasts and bone lining cells. Taken together, this suggests that osteoprogenitors, not osteocytes, are the most abundant source of RANKL. This is in contradiction with previous studies, suggesting that osteocytes are the most abundant sources of RANKL based on conditional knockout models, such as the *Dmp1-cre;Tnfsf11*^*fl/fl*^ and *Sost-cre;Tnfsf11*^*fl/fl*^ models.^27,61^ However, *Dmp1* and *Sost* promoters are not exclusively activated in osteocytes, as several lineage-tracing studies have shown that these *cre* models also result in staining of non-osteocytic cell populations, including cells on the bone surface and in the marrow.^20,22,27–29^ Our data suggests that these often-overlooked bone surface osteoprogenitor cells are likely important in the activation of osteoclastogenesis. Previously, we have established that bone lining cells, reversal cells, intracortical lumen cells, and bone marrow envelope/canopy cells express markers such as Runt-related transcription factor 2 (RUNX2), CD56, CD271, and collagen type 3 in human bone, markers characteristic to osteoprogenitors,^3,34,40,62^ as well as receptors for osteoclastic coupling factors.^63^ This supports the concept that osteoprogenitors on or above the bone surface, including bone lining cells, reversal cells, lumen cells, and bone marrow envelope cells are capable of producing RANKL and recruiting osteoclasts to the bone surface. In addition, osteoprogenitors may also respond to osteoclastic coupling factors, suggesting bidirectional communication between osteoprogenitors and osteoclasts.^63^ To characterize the cell populations expressing RANKL and OPG, we retrieved publicly available scRNAseq data of enriched bone marrow stroma isolated from mice. Interestingly, RANKL and OPG was expressed mostly by three cell clusters – in these clusters, a high proportion of RANKL^+^ cells were identified to express high levels of *Mmp13*. This was again validated in vehicle and OPG:Fc-treated mice by confirming that RANKL expressing cells on the bone surface also express RANKL. This is in line with our previous findings, where *Mmp13* has been detected at high levels in osteoprogenitor cells (i.e. reversal cells, bone lining cells and canopy cells) on the bone surfaces,^33,34^ further supporting the notion that osteoprogenitor cells on the bone surface activate osteoclastogenesis and recruit osteoclasts to the bone surfaces under physiological conditions and following withdrawal of RANKL blocking treatments.

In a recently published study, which aimed to refine the identity of mesenchymal cell types associated with murine periosteal and endosteal bone, 11 different cell clusters were identified—two of these were termed Osteo-CAR (defined by *Limch1*, *Wif1*, *Angpt4* and *Kcnk2* expression) and pre-osteoblasts (defined by *Spp1*, *Mmp13*, *Slit2*, and *Vdr* expression), respectively.^64^ Nookaew et al. confirmed the spatial organization of these cells using ISH for markers *Limch1* and *Spp1*, locating them closely to endosteal bone surfaces.^64^ In the present study, we interestingly identified three of these genes (*Limch1*, *Wif1, Mmp13*) to be highly expressed by cells that also express RANKL—further highlighting that these are osteoprogenitors. Interestingly, Nookaew et al. found that denosumab treatment of humanized RANKL mice did not have any effect on the abundance of *Limch1*^+^ Osteo-CAR cells, while it only slightly reduced the abundance of *Spp1*^+^ pre-ostebolasts.^64^ Combined with data of the present study, these findings indicate that osteoprogenitors near the bone surface play a pivotal role in the rebound resorption observed following denosumab discontinuation, which should be investigated further.

Since the time-window from the first expression of RANKL in e.g. bone lining cells, to osteoclasts are recruited to the bone surface is very narrow, it is difficult to capture the cells that first express RANKL in vivo. The recruitment of osteoclasts likely occurs within hours, as suggested by a highly increased RANKL expression in osteoblastic cells only a few hours after PTH treatment, which returns to baseline within 24 h post PTH treatment, suggesting a short-term and transient increase in RANKL upon PTH treatment.^30,31^ This aligns well with the present findings, where an upregulation of RANKL expression and downregulation of OPG expression was seen in mostly in bone surface cells and osteocytes near the bone surface—further supporting our findings in the OPG:Fc-treated mice.

The expression of OPG was observed mainly in bone surface cells (predominantly bone-forming osteoblasts), osteocytes, and fibrocytes of the rat inner ear. Moreover, the OPG expression by bone surface cells and osteocytes residing near the cortical and trabecular bone surfaces was significantly reduced upon OPG:Fc treatment in mice. This is consistent with a recent study that examined the effect of denosumab treatment in humanized RANKL mice, and reported a reduced OPG expression by cortical and trabecular osteocytes, and that sclerostin-negative osteocytes close to the bone surface are the predominant source of OPG.^20^ However, some of the OPG-expressing cells appear as bone surface cells, which may be OPG-expressing osteoblasts that are lost upon denosumab treatment, as these osteoblasts are also a source of OPG,^26^ consistent with the finding of the present study where osteoblasts were found to be an abundant source of OPG. Likewise, denosumab treatment reduces the expression of both osteoclast and bone-forming osteoblast-related genes in postmenopausal women following a single dose of denosumab,^51,65^ and bone formation parameters in osteoporotic patients treated with denosumab,^53^ as osteoblastic bone formation is normally tightly coupled to osteoclastic bone resorption during the bone remodeling process.^3,49^ These findings further support the view that bone-forming osteoblasts on the bone surface are a source of OPG, which during denosumab treatment is abolished, leading to loss of newly embedded osteocytes. Nonetheless, it is likely that loss of osteoblasts and newly formed osteocytes both – but not necessarily to a similar extent—contribute to the reduced expression of OPG near the bone surface upon blocking of RANKL.

In addition, a high OPG-expression was also observed in chondrocytes in the tibial epiphyseal growth plate and in articular cartilage, while RANKL was much less expressed in the chondrocytes and highly expressed in the primary spongiosa beneath the epiphyseal growth plate. The expression patterns of OPG in chondrocytes are in line with the findings of an older isotope-based ISH study on rats,^66^ as well as those of the recent study on a humanized RANKL murine model.^20^ On the other hand, RANKL expression is high in the primary spongiosa where, where it promotes osteoclast recruitment for resorption of the calcified cartilage and its subsequent remodeling into trabecular bone. The expression of OPG by chondrocytes in the growth plate and articular cartilage, as well as by fibrocytes lining the otic capsule of the inner ear, may serve as a protective mechanism,^20,38,66^ as abnormal bone resorption at these sites is seen in OPG deficient mice.^37,67^ Interestingly, the endogenic OPG-mediated inhibition of osteoclastic bone resorption by fibrocytes lining the inner ear does not lead to an increased abundance of RANKL-expressing cells. This implies that not all RANKL-blocking conditions lead to an accumulation of osteoclastogenic activation sites.

One limitation of this study is that we utilized ISH to detect the cells that express RANKL and OPG, which may not necessarily reflect expression at the translational level. However, previous detections of RANKL and OPG using immunohistochemical techniques, where OPG immunoreactivity has been reported in osteoblasts, pre-osteoblasts, and osteocytes^68^—whereas RANKL immunoreactivity has been reported in canopy and bone lining cells,^69,70^ support translation of mRNA at the positions corresponding with the present ISH. Secondly, we used young mice to study the cellular mechanisms leading to rebound following OPG:Fc withdrawal. Future studies should preferably investigate these mechanisms in older, and ovariectomized mice, as it has previously been shown that estrogen deficiency specifically increases RANKL expression in bone lining cells in mice.^69^ Similarly, while we utilized bone specimens from adolescents, allowing for spatiotemporal analysis of cellular events involved in the activation of osteoclastogenesis, it is essential to recognize that our findings may not generalize to other age groups. Finally, the present study utilized AI-based technology to quantify cells, which could potentially influence the accuracy of cell counts due to various factors, including variability in cell morphology and high cell densities, requiring a large training data to optimize.

In summary, we utilized multiple models to assess the local sources of RANKL and OPG. In each model, OPG appears as a protective factor acting over a broad matrix zone, whereas RANKL is responsible for a localized activation of osteoclastogenesis near the bone surfaces. We demonstrate that under physiological conditions, osteoprogenitors, including bone lining cells, reversal cells, lumen cells, and canopy cells are the main source of RANKL, while osteocytes, osteoblasts, fibrocytes lining the inner ear, and chondrocytes of the growth plate and articular cartilage are the major sources of OPG. PTH treatment in mice illustrated that bone surface cells, osteocytes and marrow cells close to the bone surface are capable responding to systemic PTH by increasing RANKL expression and decreasing OPG expression, with bone surface cells being the primary responder to this stimulus. Furthermore, we showed that OPG:Fc treatment in murine models mimics denosumab treatment in humans, and results in accumulation of osteoclastogenic activation sites with increased RANKL-expressing bone surface cells on trabecular surfaces, but not on endocortical bone surfaces. Moreover, OPG:Fc treatment results in a decreased expression of OPG in bone surface cells and osteocytes close to the bone surface, resulting in a local shift in the RANKL/OPG ratio, setting the stage for rebound resorption upon discontinuation. Further characterization of cell populations using scRNAseq of murine enriched bone marrow stroma revealed that *Tnfsf11*^+^ cells had a high expression of genes such as *Mmp13*, *Wisp2*, *Limch1, and Wif1*, and a low expression of genes such as *Col1a1, Lepr*, and *Lpl*. Multiplex ISH further confirmed the co-expression of *Mmp13* and *Tnfsf11* in bone surface cells of both vehicle and OPG:Fc-treated mice. Taken together, these findings suggest that bone surface osteoprogenitors and osteocytes conjointly regulate the activation of osteoclastogenesis in a physiological state and following denosumab discontinuation.

## Methods

### Animal experiments

#### OPG:Fc treatment of C57BL/6 J female mice

Three groups of 6–8-week-old female C57BL/6 J mice (*n* = 8/group) were obtained from Australian BioRsources and subjected to a treatment regimen with 10 mg/kg OPG:Fc (Amgen inc.), or vehicle (saline) administered i.p. thrice weekly for 2 weeks. One cohort of mice was sacrificed at the end of the 2 weeks of treatment, while three other cohorts were treated for 2 weeks followed by treatment withdrawal for 6, 9, and 11 weeks, i.e., sacrificed at week 8, 11, and 13, respectively (Fig. 1a). Bones were harvested immediately after sacrifice. Animal experiments were performed in accordance with approved protocols from the Garvan Institute/St Vincent’s Hospital Animal Ethics committee (ARA 21/17 and 18/03) and conducted in accordance with the Australian Code of Practice and Use of Animals for Scientific purposes. All animals were acclimatized upon arrival and housed under a constant temperature of 21.4 °C in ventilated cages with a 12-h light/dark cycle. Mice were provided with standard chow and water *ad libitum*.

#### Dual-energy X-ray absortiometry (DEXA)

To assess bone mineral density (BMD) in OPG:Fc- or vehicle-treated mice, DEXA was performed every two or three weeks and at the end of the study. Prior to scanning, mice were anesthetized with 3%–5% inhaled isoflurane using the Faxitron Ultrafocus DEXA system (Hologic, USA). BMD was assessed using the Vision DEXA system (Hologic, USA), with a manually delineated region of interest covering the left hind limb.

#### Micro-computed tomography (µCT)

Right femora from OPG:Fc- and vehicle-treated mice were scanned using a SkyScan 1772 µCT scanner (Bruker, Belgium). The scans were performed using a 0.5 mm aluminum filter, isotropic voxel size of 5 µm, and an X-ray voltage of 50 kV and current of 200 µA. The bone micro-architecture was examined using a 0.5-mm-high volume of interest (VOI) starting 3 mm proximal to the distal femoral growth plate and quantified using the CTAn software (Bruker, Belgium). Scans were reconstructed using SkyScan nRecon (Bruker, Belgium) and the range of attenuation coefficients used was between 0.0 and 0.12. The µCT images were segmented using a global threshold value of 792 mg HA/cm^3^. The trabecular analysis included: trabecular thickness, trabecular separation, and trabecular bone volume/tissue volume (BV/TV). Additional analyses included cortical bone, and marrow space cross-sectional areas.

#### PTH treatment of C57BL/6 J female mice

12-week-old female C57BL/6 J mice (Jackson Laboratories, strain 00664) were subjected to a single dose of 80 µg/kg PTH (1–34) (Bachem), or vehicle (saline) administered i.p. (n = 3/group). Mice were sacrificed 1 h following PTH injection and femora were harvested. Animal experiments were performed in accordance with approved protocols from the institutional Animal Care and Use Committee of Harvard Medical School (2016N000303) and conducted in accordance with relevant guidelines ad laws. All animals were acclimatized upon arrival and housed under a constant temperature of 21.4 °C in ventilated cages with a 12-h light/dark cycle. Mice were provided with standard chow and water *ad libitum*.

#### C57B6 female and male mice

12-week-old female and male C57BL/6 J mice (*n* = 5/gender) were obtained from Janvier Labs. Animals were sacrificed, and vertebrae were harvested. Animal experiments were performed in accordance with approved protocols from the Danish Ethical Committee for Animal Studies (2018-15-0201-01436). All animals were acclimatized upon arrival and housed under constant temperature of 21 °C in ventilated cages with a 12-h light/dark cycle. Mice were provided with standard chow and water *ad libitum*.

#### Sprague-Dawley rats

10-day-old male Sprague-Dawley rats (*n* = 4) were obtained from Charles River. Animals were first acclimatized and then sacrificed, followed by harvest of the crania, harboring the inner ear. Animal experiments were approved by the Danish Ethical Committee for Animal Studies (P-19-315). All animals were housed under a constant temperature of 22 °C in ventilated cages with a 12-h light/dark cycle. Rats were provided with standard chow and water *ad libitum*.

### Human bone specimen

Human cortical bone specimens were obtained from the proximal femur of 4 healthy adolescent females (aged 14–16 years) undergoing corrective surgery for Coxa Valga, a deformity of the hip. The study was approved by the Danish National Committee on Biomedical Research Ethics (Project ID: S-2012- 0193). 3-mm Jamshidi Iliac crest bone biopsies was obtained from 2 healthy females (aged 33 and 49) and 2 males (aged 27 and 41). The study was approved by the Danish National Committee of Biomedical Research Ethics (Project ID S-20110112). Written informed consent was received prior to participation in this study.

### Tissue preparation

All tissue samples were fixed immediately in 4% paraformaldehyde for 24–48 h, decalcified in 0.5 mol·L^−1^ EDTA, paraffin-embedded, serially sectioned into 3.5-µm-thick sections. All sections were stored at 4 °C before the subsequent experiments.

### Masson trichrome stain

Every 5th human bone section was Masson trichrome stained as previously described^3,62^ to select sections with active intracortical bone remodeling. Singleplex ISH was performed on an adjacent section from each individual, as described below.

### Chromogenic ISH combined with immunostaining

Singleplex chromogenic ISH was performed using a modified version of the RNAScope 2.5 HD procedure (310035, Advanced Cell Diagnostics [ACD]). Sections were deparaffinized in xylene, dehydrated in 99% ethanol, and blocked with 3% hydrogen peroxide for 30 min at room temperature. Target retrieval and nuclei permeabilization was performed by incubating samples in ACD’s Target Retrieval Reagent (Catalog 322000) in an 80–90 °C water bath, followed by treatment with 10%–20% pepsin in diethylpyrocarbonate- (DEPC-) treated water. Subsequently, sections were probe hybridized overnight at 40 °C using 20 double-Z probe pairs with complementary RNA to the target mRNA. Tissue sections were incubated with following probes diluted 1:1 in probe diluent: mouse *Acp5* (Mm-Acp5, catalog 465001), mouse *Tnfsf11* (Mm-Tnfsf11, catalog 410921), mouse *Tnfrsf11b* (Mm-Tnfrsf11b, catalog 488961), rat *Tnfsf11* (Rn-Tnfsf11, catalog 813111), rat *Tnfrsf11b* (Rn-Tnfrsf11b, catalog 813091), human *TNFSF11* (Hs-TNFSF11, catalog 412271), and human *TNFRSF11B* (Hs-TNFRSF11B, catalog 412291). Following probe hybridization, a 6-step branch amplification was carried out according to the manufacturer’s instructions. The signal was further amplified using digoxygenin (DIG)-labeled tyramide from a tyramide signal amplification (TSA) plus DIG Reagent kit (NEL748001KT, PerkinElmer). Sections were washed in TBS/Tween and incubated with Anti-DIG FAB fragments conjugated to alkaline phosphatase (11093274910, Roche). The mRNA signal was developed using Liquid Permanent Red (K064030-2, Agilent).

To detect mature osteoclasts in human bone, sections were further stained for TRAcP using immunohistochemistry. Tissue sections were first washed in demineralized water, blocked in casein diluted in TBS, followed by incubation with a primary mouse IgG2b anti-tartrate-resistant acid phosphatase (TRAcP) antibody (clone 9C5, MABF96, Merck Millipore). Subsequently, sections were washed in TBS, blocked in 3% hydrogen peroxide in TBS, and incubated with horseradish peroxidase (HRP)-conjugated secondary anti-mouse antibody (BrightVision, Immunologic). The sections were washed in TBS and the signal was developed using Deep Space Black (BC-BRI4015H, HISTOLAB). All sections were counterstained with Mayer’s hematoxylin, and mounted in Aquatex. Slides were scanned using an Olympus VS200 Slidescanner or a Hamamatsu slide scanner.

### Singleplex and multiplex fluorescence ISH

Singleplex fluorescent ISH was performed using a modified version of the RNAscope 2.5 high-definition procedure (322310, ACD), on sectioned tibiae harvested from vehicle and OPG:Fc-treated mice. The staining was performed similarly to the chromogenic ISH with minor modifications. In short, sections were deparaffinized in xylene, blocked with 3% hydrogen peroxide, followed by target retrieval and tissue permeabilization using a custom pretreatment reagent (catalog 300040). Sections were hybridized using 20 double-Z probe pairs targeting mouse *Acp5* (Mm-Acp5, catalog 465001), mouse *Tnfsf11* (Mm-Tnfsf11, catalog 410921), mouse *Tnfrsf11b* (Mm-Tnfrsf11b, catalog 488961). Following hybridization, branch amplification was carried out according to the manufacturer’s instructions and the signal was developed with HRP followed by incubation with the fluorochrome Opal 570.

Multiplex fluorescent ISH was performed using a modified version of the RNAscope Multiplex Flourecent Assay (323110, ACD). Tissue sections were deparaffinized and pretreated as in the singleplex ISH. Following, sections were hybridized using multiplexing probes simultaneously. The probes consisted of 20 double-Z probe pairs with three distinct spectral channels (C), targeting mouse *Tnfsf11* (C1-Mm-Tnfsf11, catalog 410921) or mouse *Tnfrsf11b* (C1-Mm-Tnfrsf11b, catalog 488961) in combination with mouse *Mmp13* (C3-Mm-Mmp13, catalog 427601, ACD) and mouse *Col1a1* (C4-Mm-Col1a1, catalog, 319371ACD). Following probe hybridization, a 3-step branch amplification was carried out according to the manufacturer’s instructions. The signal was developed using sequential TSA-enhancement with fluorochrome Opal 570 for channel 1, Opal 620 for channel 3 and Opal 690 for channel 4. Tissue sections were blocked using a hydrogen peroxidase blocker followed by incubation with a new peroxidase-conjugated probe for the subsequent channel. In singleplex- and multiplex-stained sections, nuclei in were stained using 2.5 µg/mL Hoechst (H3569, Invitrogen) and mounted in Prolong gold antifade reagent (P36939, Invitrogen). Slides were scanned using an Olympus VS200 slide scanner.

### Quantification and spatial analysis using HALO AI

AI-based quantification and spatial analysis was performed using HALO and HALO AI (Indica Labs) on tissue sections of the tibia collected from OPG:Fc- and vehicle-treated mice. Three regions of interest (ROIs) were defined on all scanned sections. The first ROI was defined as a 3-mm-long region, and included the marrow and the trabecular bone compartment, while the cortical bone, growth plate, and primary spongiosa were excluded. In the vehicle-treated mice, measurements began 400 µm distal to the proximal epiphyseal growth plate, while in the OPG:Fc-treated mice, measurements began 600 µm distal to the proximal epiphyseal growth plate, due to the increased length of the primary spongiosa in OPG:Fc-treated mice (Fig. S8a). The nuclei were quantified on a single cell level using a pre-trained deep-learning-based neuronal network (HALO AI, Indica Labs), which was improved on these sections by marking approximately 3 000 additional nuclei before the analysis. Each cell’s mRNA staining intensity of either *Tnfsf11* or *Tnfrsf11b* mRNA was quantified using HALO’s thresholding parameters, which were determined using the real-time tuning function. All parameters were held constant in-between samples. Following nuclei quantification, a spatial analysis was conducted by first drawing an interface line on all trabecular bone surfaces. The distance of every single cell from the interface line was measured up to 20 µm into the marrow 50 µm into the trabecular bone. Surface cells were defined as cells that were less than 3 µm above or below the interface line, while marrow cells were defined as cells more than 3 µm above the interface line and osteocytes as cells more than 3 µm below the interface line. A second 2.5-mm-long ROI was defined, encompassing both cortical compartments, and the marrow compartment. The ROI started 1 mm distal to the proximal epiphyseal growth plate and extended 2.5 mm in both groups. The primary spongiosa, trabecular bone, periosteum, and transcortical canals were excluded from the analysis, to focus on the endocortical surface cells and cortical osteocytes (Fig. S8b). The number of nuclei and *Tnfsf11* and *Tnfrsf11b* mRNA staining intensities were quantified and a spatial analysis was performed by first drawing an interface line, outlining the endocortical bone surfaces of both cortices, and measuring the distance of each cell from the interface line and up to 500 µm into the marrow and 300 µm into the cortical bone. Finally, a third ROI encompassing the growth plate and the primary spongiosa was drawn and the nuclei and their *Tnfsf11* and *Tnfrsf11b* mRNA intensity was quantified. Following, a spatial analysis was performed, where the single cell distances up to 200 µm above or below an interface line, separating the primary spongiosa from the growth plate were measured (Fig. S8c).

The same spatial analysis pipeline was employed to analyze the *Tnfsf11* and *Tnfrsf11b* expression in PTH-treated mice, as in OPG:Fc-treated mice. In short, AI-based quantification and spatial analysis was performed using HALO and HALO AI (Indica Labs) on tissue sections of the femur collected from PTH- and vehicle-treated mice. For first ROI encompassing the trabecular and marrow compartment, the ROI started 400 µm proximal to the distal femoral growth plate and extended 4 mm (Fig. S9a). For the ROI encompassing both cortical bone compartments and the marrow compartment, the analysis started 1 mm proximal to the distal femoral and extended 5 mm (Fig. S9a).

### Manual quantification and spatial analysis of chromogenic/Fluorescent ISH

#### Vehicle and OPG:Fc-treated mice

Osteoclast perimeter and total bone perimeters were manually measured in HALO (HALO AI, Indica Labs) based on *Acp5* ISH to determine trabecular and endocortical Oc.Pm/B.pm in vehicle and OPG:Fc-treated mice at week 2, 11, and 13. All perimeters were measured in the previously defined ROIs employed in the *Tnfsf11*/*Tnfrsf11* spatial analysis.

#### Human bone specimens

The percentage of *TNFSF11*^+^ and *TNFRSF11B*^+^ cells in adjacent human femoral bone sections was calculated by manually counting *TNFSF11*^+^, *TNFRSF11B*^+^, and negative cells within a distance of 25 µm from TRAcP^+^ surface osteoclasts and lumen osteoclasts. The quantified cells were included matrix-embedded osteocytes, mononuclear reversal cells on the eroded bone surface, mononuclear lumen cells, and endothelial cells of vascular structures. Collectively, these cells were defined as peri-osteoclastic cell populations, residing next to osteoclasts. We further quantified the number of positive and negative osteocytes residing 25–100 µm away from surface osteoclasts. Finally, we quantified the number of positive and negative osteoblasts on osteoid surfaces and bone lining cells on quiescent bone surfaces – these cells were defined as cell populations residing away from osteoclasts. The different types of surfaces, including eroded, osteoid and quiescent surfaces, were validated using an adjacent Masson trichrome stained section.

#### Mouse vertebral bone

Paraffin-embedded lumbar vertebrae (L3–L4) were sectioned in the coronal plane. Subsequently, adjacent sections were stained for *Tnfsf11*, *Acp5*, or *Tnfrsf11b* mRNA using singleplex chromogenic ISH as described above. A ROI was defined by measuring 350 µm from both growth plates of L3 or L4, depending on the tissue quality, encompassing the trabecular and endocortical bone compartment (Fig. S9b). The percentage of *Tnfsf11*^+^ and *Tnfrsf11*^+^ cells was quantified by first identifying *Acp5*^+^ osteoclasts on the bone surfaces of one section, followed by localization of the osteoclast on the adjacent section stained for *Tnfsf11* or *Tnfrsf11*. Following, *Tnfsf11*^+^, *Tnfrsf11b*^+^, and negative cells that were up to 25 µm away from osteoclasts residing on trabecular or endocortical bone surfaces were manually quantified; collectively, these were defined as cells next to osteoclasts. Osteocytes further away from osteoclasts, were defined as cells located more than 25 µm away from an osteoclast.

### Bone marrow scRNA-seq bioinformatic analysis

Mouse bone marrow scRNAseq datasets (accession number GSM3674243, GSM3674244, GSM3674245, and GSM3674246) were downloaded from the NCBI GEO database.^32^ Seurat (v.5.0.0) was used to analyze the raw count matrix.^71^ Following a standard quality analysis and prefiltering step, gene expression for each cell was normalized by the total expression, multiplied by 10 000, and log transformed. The top 2 000 variable genes were selected by the FindVariableGenes function. A linear transformation was applied using ScaleData function followed by dimensional reduction analysis. Principal Component Analysis (PCA) was performed using the RunPCA function, followed by Uniform Manifold Approximation and Projection (UMAP) algorithm using the RunUMAP function. Cell cluster biomarkers were identified using the FindMakers function. Cell cluster identity was assigned based on published literature^32^ and the Cell Taxonomy Database (https://ngdc.cncb.ac.cn/celltaxonomy/). Genes of interest were visualized as violin plots using the VlnPlot function. FindMakers function was also applied to identify differentially expressed genes (DEGs) between *Tnfsf11*^+^ and *Tnfsf11*^-^ cells using Wilcoxon Rank Sum test. *P* value was adjusted by Bonferroni correction. DEGs meeting the criteria of adjusted *P* value less than 0.05 and log_2_ fold change (log_2_FC) higher than 0.5 were considered significant and visualized by dot plots using the DotPlot function.

### Statistics

One-way ANOVAs, two-way ANOVAs, multiple comparisons test, unpaired, and paired t-tests were conducted in GraphPad Prism V8 (GraphPad Software, Inc., La Jolla, CA, USA). One-way ANOVA and comparison tests were conducted using Holm-Sidak’s multiple comparisons test. Two-way ANOVA and comparison tests were conducted using either Sidak’s multiple comparisons test or Tukey’s multiple comparisons test. Mixed-effects analysis was conducted using Sidak’s multiple comparisons test. In the figures, data are expressed as either means with error bars representing standard deviation or means with error bars representing standard error of the mean. *P* values < 0.05 were considered statistically significant. Clustered logistic regressions were conducted using STATA (STATA 18, StataCorp, College Station, TX, USA) and were utilized to calculate odds ratios (ORs), i.e., the likelihood of a greater or smaller prevalence of a given factor in one cell population compared with another cell population. An OR greater than one indicates a higher prevalence of a given factor in one cell population compared to another, while an OR smaller than one indicates a lower prevalence. Human data included observations from 4 biopsies treating each donor as a cluster. ORs, confidence intervals, and statistical significance were calculated by using the upstream cell population as a reference. The prevalence of a given factor was visualized in histograms illustrating, the percentage of cells positive for selected genes.

## Supplementary information

Supplementary information

Table S1

Figure S1

Figure S2

Figure S3

Figure S4

Figure S5

Figure S6

Figure S7

Figure S8

Figure S9

## Data Availability

The data sets generated in the present study are available from the corresponding author upon reasonable request.
