# Supplementary material for: Mapping RANKL- and OPG-expressing cells in bone tissue: the bone surface cells as activators of osteoclastogenesis and promoters of the denosumab rebound effect
Source: Bone Res. 2024 Oct 18;12:62. doi: 10.1038/s41413-024-00362-4 (PMC11489716; doi:10.1038/s41413-024-00362-4)
Supplement: Supplementary file 1 — Supplementary information [file 41413_2024_362_MOESM1_ESM.docx]

**Figure S1 – Supplementary µCT analysis of vehicle vs OPG:Fc-treated mice. a** Trabecular thickness, **b** trabecular separation, and **c** total cross-sectional area at 2, 8, 11, and 13 weeks. **a-g** Data are expressed as mean ± SD. *P* values are calculated using an unpaired t-test. *****P* values < 0.0001, ****P* values < 0.001, ***P* values < 0.01, **P* values < 0.05.*n* = 7-10/group.

**Figure S2 – Endocortical surfaces are occupied by less osteoclasts at week 13 after OPG:Fc withdrawal.** The graphs compare trabecular and endocortical Oc.Pm/B.Pm in vehicle and OPG:Fc-treated mice at week 2, 11, and 13. Data are expressed as mean ± SD. *P* values are calculated using a paired *t*-test. ***P* values < 0.01, **P* values < 0.05.*n* = 3/group. Abbreviations: Trabecular (T), endocortical (E), Osteoclast perimeter/Bone perimeter (Oc.Pm/B.Pm).

**Figure S3** – **The effect of OPG:Fc treatment on *Tnfsf11* and *Tnfrsf11b* expression in trabecular and cortical bone. a** Mean *Tnfsf11* staining intensity against the distance from the trabecular or endocortical bone surface. **b** Mean *Tnfrsf11b* staining intensity against the distance from the trabecular or endocortical bone surface. **c** *Tnfsf11/Tnfrsf11b* positive cell ratios and staining intensity ratios in trabecular surface cells, trabecular osteocytes, and proximate marrow cells. **d** *Tnfsf11/Tnfrsf11b* positive cell ratios and staining intensity ratios in endocortical surface cells, cortical osteocytes and marrow cells. **a, b** Data are expressed as mean ± SEM. **c-d** Data are expressed as mean ± SD. all *P* values are calculated using a two-way ANOVA. *****P* values < 0.0001, ****P* values < 0.001, ***P* values < 0.01, **P* values < 0.05. *n* = 8/group.

**Figure S4 – The effect of OPG:Fc treatment on *Tnfsf11* and *Tnfrsf11b* expression in the epiphyseal growth plate of the tibia. a** *Tnfsf11/Tnfrsf11b* positive cell ratios and staining intensity ratios in chondrocytes and cells of the primary spongiosa. **b** Mean *Tnfsf11* and **c** *Tnfrsf11b* staining intensity against the distance from the border between the primary spongiosa and chondrocytes. **a** Data are expressed as mean ± SD. **b,c** Data are expressed as mean ± SEM. *P* values are calculated using a two-way ANOVA or a mixed-effects analysis. *****P* values < 0.0001, ***P* values < 0.01, **P* values < 0.05. *n* = 8/group.

**Figure S5 – Supplementary single cell RNAseq data. a** Mouse single cell RNAseq dataset info. **b** Key gene markers defining identity bone marrow cell clusters of interest (0) Lepr^+^ Bglap^+^ mesenchymal cell (MSC), (3) Lepr^+^ Bmp4^hi^ MSC, and (7) osteolineage cell (Osteo).

**Figure S6 – The effect of a single dose of PTH treatment on *Tnfsf11* and *Tnfrsf11b* expression in trabecular and cortical bone.** All data is from ISH conducted on sections of the femur from mice treated with PTH or vehicle**. a** Mean *Tnfsf11* staining intensity against the distance from the trabecular or endocortical bone surface. **b** Mean *Tnfrsf11b* staining intensity against the distance from the trabecular or endocortical bone surface. **c** *Tnfsf11/Tnfrsf11b* positive cell ratios and staining intensity ratios in trabecular surface cells, trabecular osteocytes, and proximate marrow cells. **d** *Tnfsf11/Tnfrsf11b* positive cell ratios and staining intensity ratios in endocortical surface cells, cortical osteocytes and marrow cells. **a, b** Data are expressed as mean ± SEM. **c, d** Data are expressed as mean ± SD. *P* values are calculated using a two-way ANOVA or a mixed-effects analysis. *****P* values < 0.0001, ****P* values < 0.001, ***P* values < 0.01, **P* values < 0.05. *n* = 3/group.

**Figure S7 – Male and female mice exhibit a similar expression pattern of *Tnfsf11* and *Tnfrsf11b*.** All data is from ISH conducted on vertebral bone sections from a 12-week-old male and female mice. **a, b** Histograms illustrating the mean percentage of *Tnfsf11*^+^, or *Tnfrsf11b*^+^ cells, respectively. All data are shown as mean ± SD and *P* values are calculated using a two-way ANOVA. *****P* values < 0.0001, ****P* values < 0.001, ***P* values < 0.01, **P* values < 0.05. *n* = 5/group. Scale bars: = 50 µm. Abbreviations: Trabecular (Tb), bone marrow (Bm).

**Table S1–*TNFRSF11* and *TNFRSF11B* odds ratios calculated from different cell populations from human femoral bone sections.** Odds ratios were calculated using a clustered logistic regression, with the most upstream cell population as a reference. An odds ratio above one indicates a greater likelihood of detecting either *TNFSF11* or *TNFRSF11B* compared to the reference cell population while an odds ratio below 1 indicates a lower likelihood of detecting either *TNFSF11* or *TNFRSF11B* compared to the reference. Green indicates cell populations next to surface osteoclasts, yellow indicates cell populations next to lumen osteoclasts, while red indicates cell populations away from osteoclasts. Abbreviations: Reversal cells (Rv.Cs), Lumen cells (Lum.Cs), Endothelial cells (Ecs), Osteocytes (Ocy), Bone lining cells (BLCs), Osteoblasts (OBs).

**Figure S8 – ROIs defined in tissue sections collected from vehicle- or OPG:Fc-treated mice. a** Longitudinal section of tibiae, with a 3-mm-long ROI of the trabecular and marrow compartment, starting 400 or 600 µm distal to the proximal epiphyseal growth plate in vehicle-treated mice or OPG:Fc-treated mice, respectively. **b** Longitudinal section of tibiae, with a 2-mm-long ROI of the cortical and marrow compartment, starting 1 mm distal to the proximal epiphyseal growth plate in vehicle-treated mice, or OPG:Fc-treated mice, respectively. **c** Longitudinal section of a tibia with a defined ROI extending 200 above and below a line separating the chondrocytes from the primary spongiosa.

**Figure S9 – ROIs defined in femoral tissue sections collected from vehicle-treated mice, PTH-treated mice, and vertebral sections from non-treated mice. a** Longitudinal section of a femur with a 4-mm-long ROI of the trabecular and marrow compartment, starting 400 µm proximal to the distal epiphyseal growth plate, and a 5-mm-long ROI of the cortical and marrow compartment starting 1 mm proximal to the distal epiphyseal growth plate. ROIs are identical between vehicle- and PTH-treated mice. **b** Coronal section of a vertebra, with ROI starting 350 µm distal to both growth plate and encompassing the marrow, endocortical, and trabecular bone compartment.
