# Supplementary material for: Mapping RANKL- and OPG-expressing cells in bone tissue: the bone surface cells as activators of osteoclastogenesis and promoters of the denosumab rebound effect
Source: Bone Res. 2024 Oct 18;12:62. doi: 10.1038/s41413-024-00362-4 (PMC11489716; doi:10.1038/s41413-024-00362-4)
Supplement: Supplementary file 2 — Table S1 [file 41413_2024_362_MOESM2_ESM.pdf]

Table S1

TNFSF11					
Test		Odds ratio	Std. err.	P value	95% conf. Interval
RV.Cs	Lum. Cs	1.26454	0.336379	0.378	[0.7507677-2.129903]
RV.Cs	ECs	0.3288762	0.336379	0.018	[0.1308418-0.8266439]
RV.Cs	Ocy	0.0724326	0.0406126	0.000	[0.0241362-0.2173692]
RV.Cs	Lum.Cs	1.746248	0.5151597	0.059	[0.979475-3.113282]
RV.Cs	ECs	0.2464438	0.1913465	0.071	[0.0538045-1.1288]
RV.Cs	Ocy	0.1243821	0.0350191	0.000	[0.0716319-0.2159777]
RV.Cs	OBs	0.4416937	0.1180455	0.002	[0.2615966-0.7457795]
RV.Cs	BLCs	0.0579029	0.0218793	0.000	[0.0276098-0.1214331]
Lum.Cs	ECs	0.2639473	0.1114434	0.002	[0.1153783-0.6038236]
Lum.Cs	Ocy	0.0576148	0.0299759	0.000	[0.0207812-0.1597342]
Lum.Cs	Lum.Cs	1.370157	0.287042	0.133	[0.9087562-2.065825]
Lum.Cs	ECs	0.1947344	0.145626	0.029	[0.0449675-0.8433091]
Lum.Cs	Ocy	0.0987696	0.0186564	0.000	[0.068209-0.1430227]
Lum.Cs	OBs	0.350422	0.058545	0.000	[0.2525696-0.486185]
Lum.Cs	BLCs	0.0454299	0.0143204	0.000	[0.0244922-0.0842668]
ECs	Ocy	0.2256335	0.1464749	0.022	[0.0632161-0.8053407]
ECs	Lum.Cs	5.494642	2.464857	0.000	[2.280848-13.23678]
ECs	ECs	0.7704825	0.6507584	0.758	[0.147173-4.033642]
ECs	Ocy	0.3843331	0.1654214	0.026	[0.165326-0.8934591]
ECs	OBs	1.408928	0.6065668	0.426	[0.6059436-3.276009]
ECs	BLCs	0.179937	0.0906018	0.001	[0.0670693-0.4827444]
Ocy	Lum.Cs	24.10387	12.95275	0.000	[8.407654-69.10332]
Ocy	ECs	3.398891	3.04619	0.172	[0.5867652-19.68838]
Ocy	Ocy	1.706046	0.9008775	0.312	[0.6060574-4.802503]
Ocy	OBs	6.194189	3.243476	0.000	[2.219566-17.28625]
Ocy	BLCs	0.7923106	0.4643061	0.691	[0.251234-2.498691]
Lum.Cs	ECs	0.1428627	0.1083057	0.010	[0.0323306-0.6312827]
Lum.Cs	Ocy	0.0728135	0.0164472	0.000	[0.0467673-0.1133657]
Lum.Cs	OBs	0.2572151	0.0535935	0.000	[0.1709778-0.3869487]
Lum.Cs	BLCs	0.0331994	0.0112255	0.000	[0.0171127-0.0644084]
ECs	Ocy	0.4959857	0.3742477	0.353	[0.1130296-2.176437]
ECs	OBs	1.929688	1.449975	0.382	[0.4424772-8.415566]
ECs	BLCs	0.2322987	0.1848443	0.067	[0.0488349-1.105002]
Ocy	OBs	3.887865	0.7775256	0.000	[2.627128-5.753619]
Ocy	BLCs	0.4690561	0.1547334	0.022	[0.2457131-0.8954087]
OBs	BLCs	0.1122159	0.0363349	0.000	[0.0594895-0.2116747]

TNFRSF11B					
Test		Odds ratio	Std. err.	P value	95% conf. Interval
RV.Cs	Lum. Cs	1.491939	0.8653731	0.490	[0.4786591-4.650243]
RV.Cs	ECs	-	-	-	-
RV.Cs	Ocy	11.02093	6.178996	0.000	[3.672681-33.07143]
RV.Cs	Lum.Cs	0.5544842	0.401298	0.415	[0.1342293-2.290504]
RV.Cs	ECs	-	-	-	-
RV.Cs	Ocy	3.058941	1.607959	0.033	[1.091766-8.570626]
RV.Cs	OBs	6.345376	3.328076	0.000	[2.269928-17.73792]
RV.Cs	BLCs	1.135672	0.6557492	0.826	[0.366235-3.521647]
Lum.Cs	ECs	-	-	-	-
Lum.Cs	Ocy	7.37322	2.543703	0.000	[3.749724-14.49823]
Lum.Cs	Lum.Cs	0.3700772	0.2134806	0.085	[0.1194739-1.146335]
Lum.Cs	ECs	-	-	-	-
Lum.Cs	Ocy	2.038295	0.5867853	0.013	[1.159372-3.583533]
Lum.Cs	OBs	4.252818	1.219919	0.000	[2.423874-7.461801]
Lum.Cs	BLCs	0.7657763	0.2838038	0.471	[0.3703725-1.583307]
ECs	Ocy	-	-	-	-
ECs	Lum.Cs	-	-	-	-
ECs	ECs	-	-	-	-
ECs	Ocy	-	-	-	-
ECs	OBs	-	-	-	-
ECs	BLCs	-	-	-	-
Ocy	Lum.Cs	0.0479868	0.0268269	0.000	[0.0160421-0.1435435]
Ocy	ECs	-	-	-	-
Ocy	Ocy	0.2710953	0.066285	0.000	[0.1678791-0.4377716]
Ocy	OBs	0.5662004	0.1390795	0.021	[0.3498517-0.9163394]
Ocy	BLCs	0.1039066	0.0350414	0.000	[0.0536519-0.2012341]
Lum.Cs	ECs	-	-	-	-
Lum.Cs	Ocy	5.512661	2.877694	0.001	[1.981623-15.33563]
Lum.Cs	OBs	11.52593	6.005605	0.000	[4.151032-32.00339]
Lum.Cs	BLCs	2.087785	1.202795	0.201	[0.6749936-6.45761]
ECs	Ocy	-	-	-	-
ECs	OBs	-	-	-	-
ECs	BLCs	-	-	-	-
Ocy	OBs	2.098781	0.3189276	0.000	[1.558188-2.826926]
Ocy	BLCs	0.3873222	0.1095315	0.001	[0.2225152-0.6741944]
OBs	BLCs	0.1375931	0.0377186	0.000	[0.0803999-0.2354712]
