# Supplementary figures and images for: Mapping RANKL- and OPG-expressing cells in bone tissue: the bone surface cells as activators of osteoclastogenesis and promoters of the denosumab rebound effect
Source: Bone Res. 2024 Oct 18;12:62. doi: 10.1038/s41413-024-00362-4 (PMC11489716; doi:10.1038/s41413-024-00362-4)

Figure S1

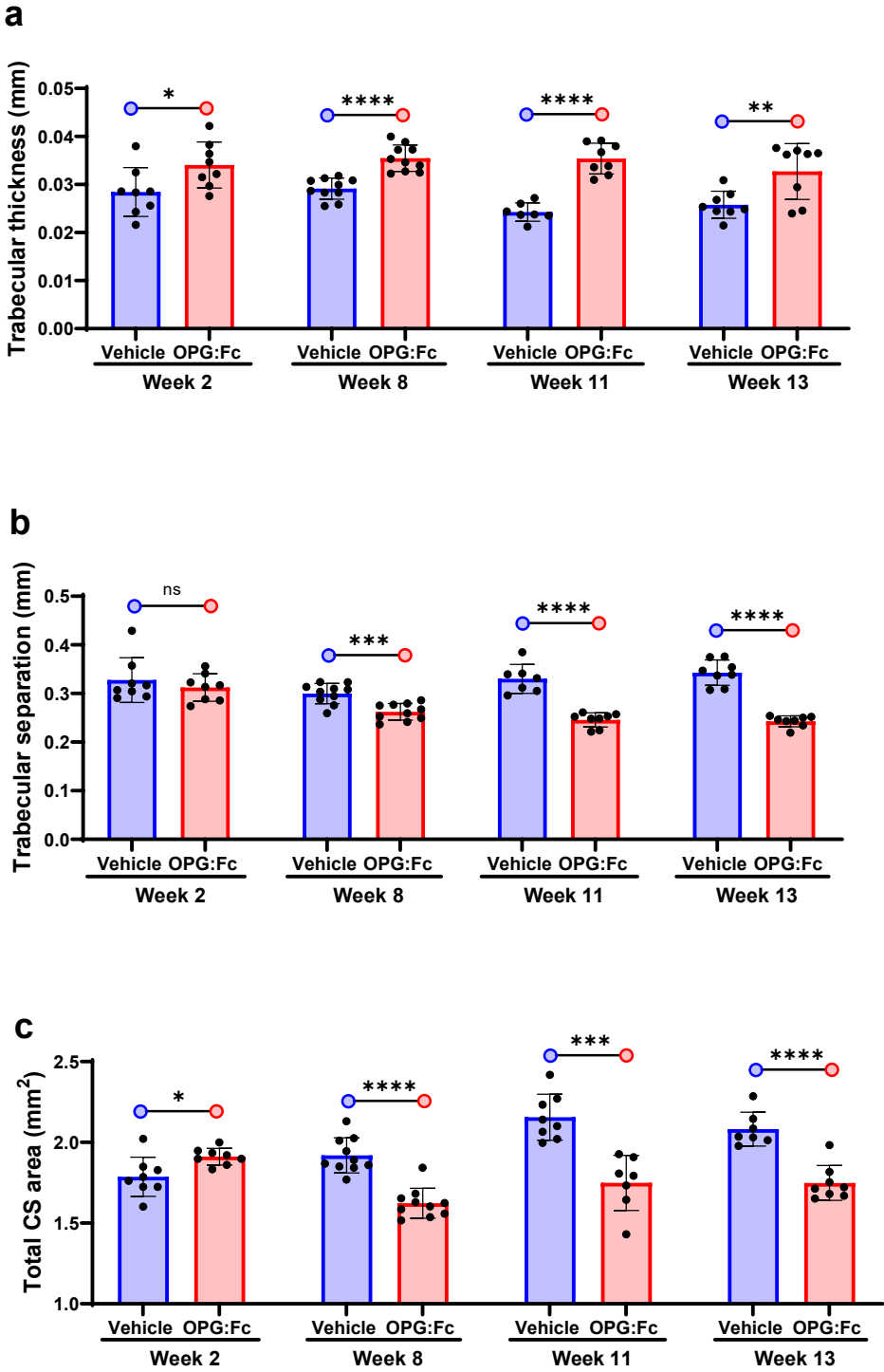

Supplement: Supplementary file 3 — Figure S1 [file 41413_2024_362_MOESM3_ESM.pdf]

Figure S2

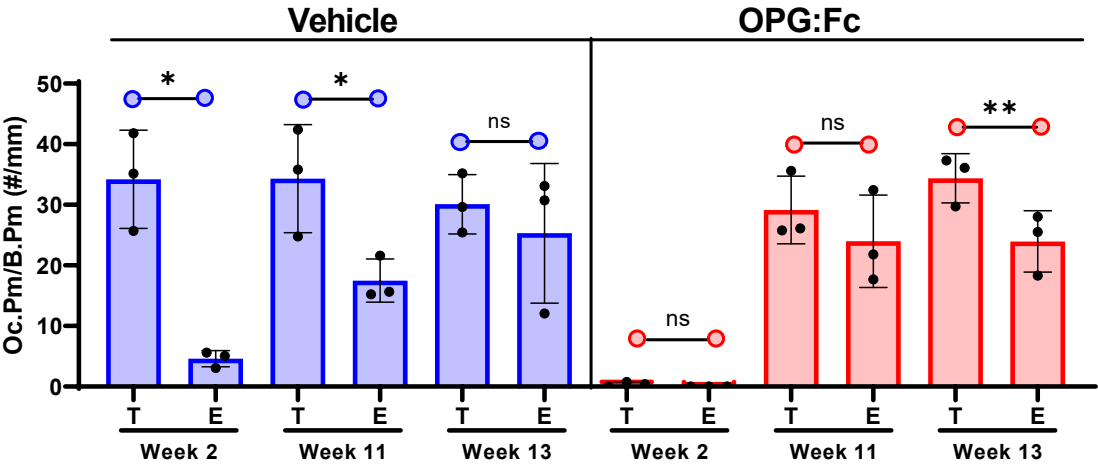

Supplement: Supplementary file 4 — Figure S2 [file 41413_2024_362_MOESM4_ESM.pdf]

Figure S3

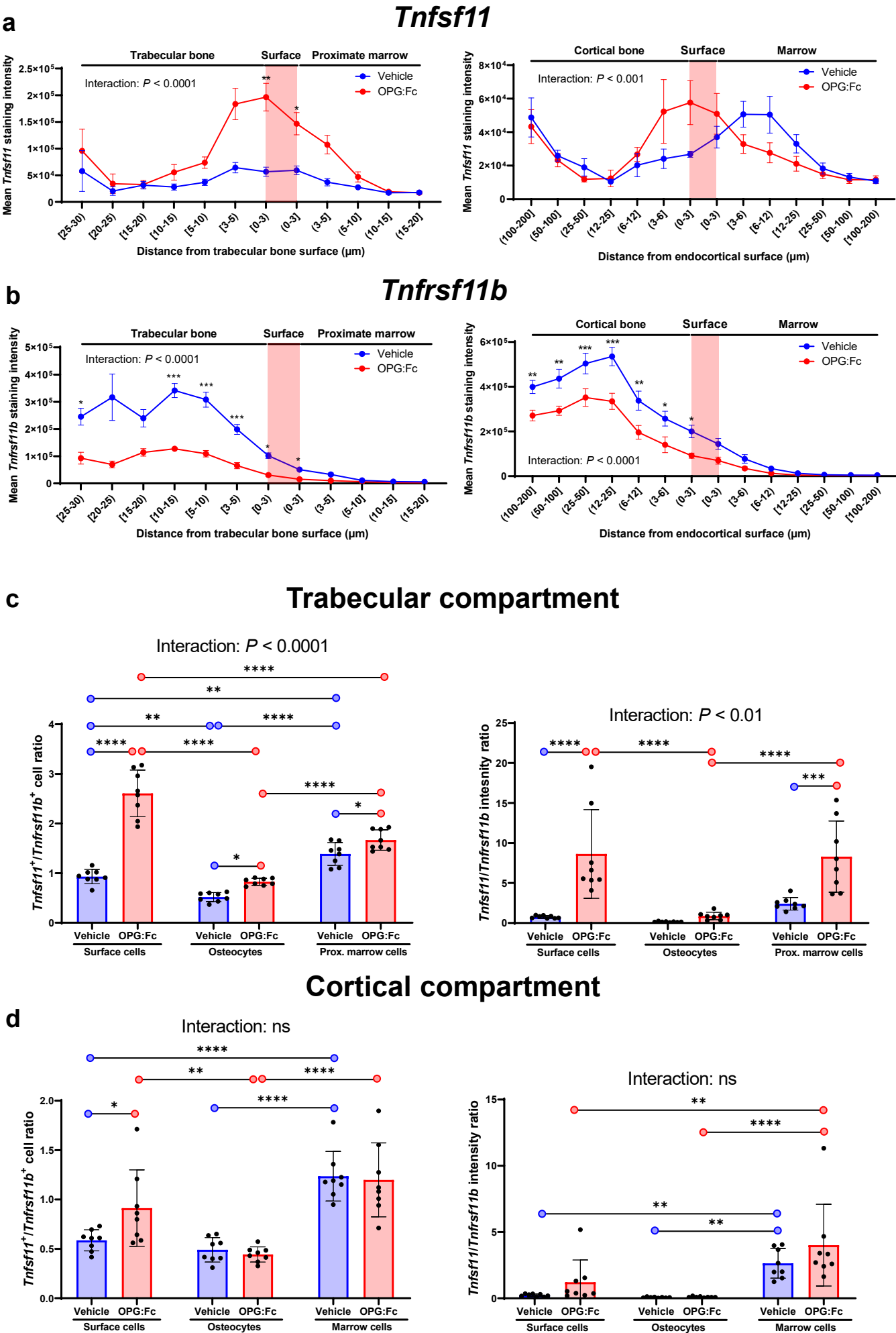

Supplement: Supplementary file 5 — Figure S3 [file 41413_2024_362_MOESM5_ESM.pdf]

Figure S4

Growth plate

a

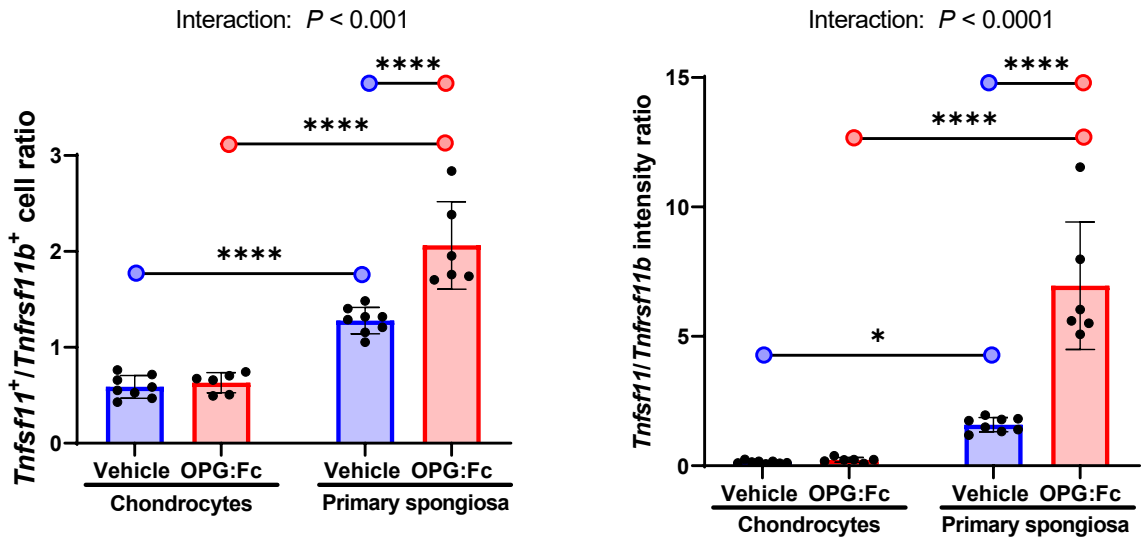

b

*Tnfsf11*

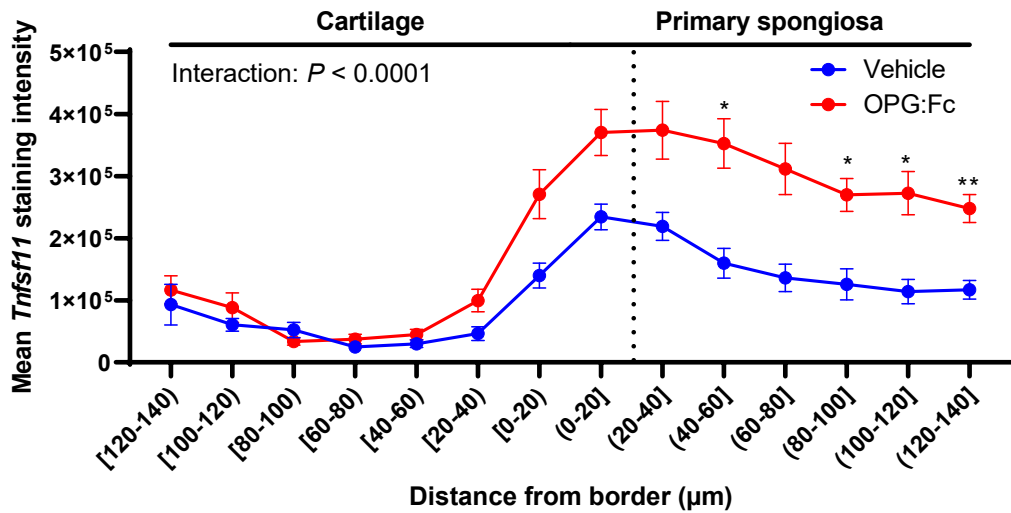

c

*Tnfrsf11b*

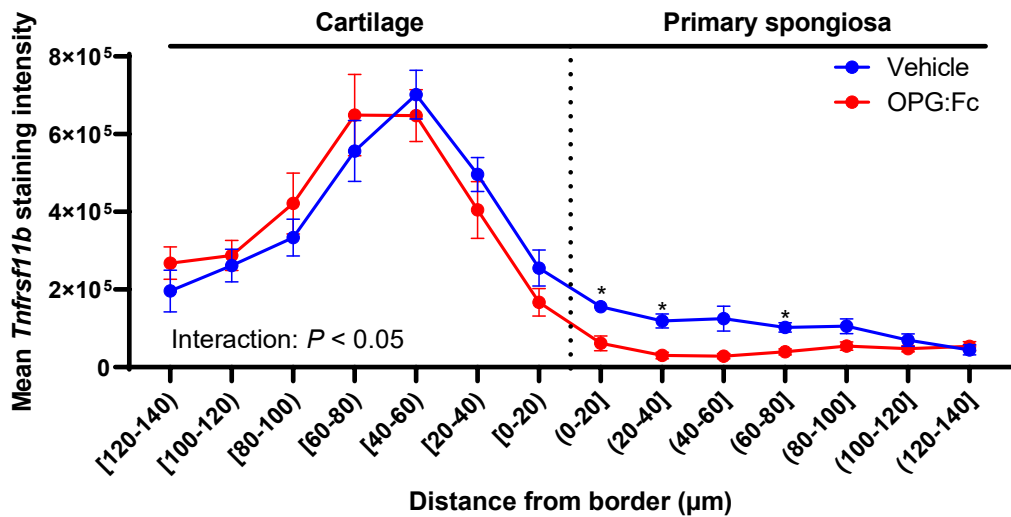

Supplement: Supplementary file 6 — Figure S4 [file 41413_2024_362_MOESM6_ESM.pdf]

Figure S5

a

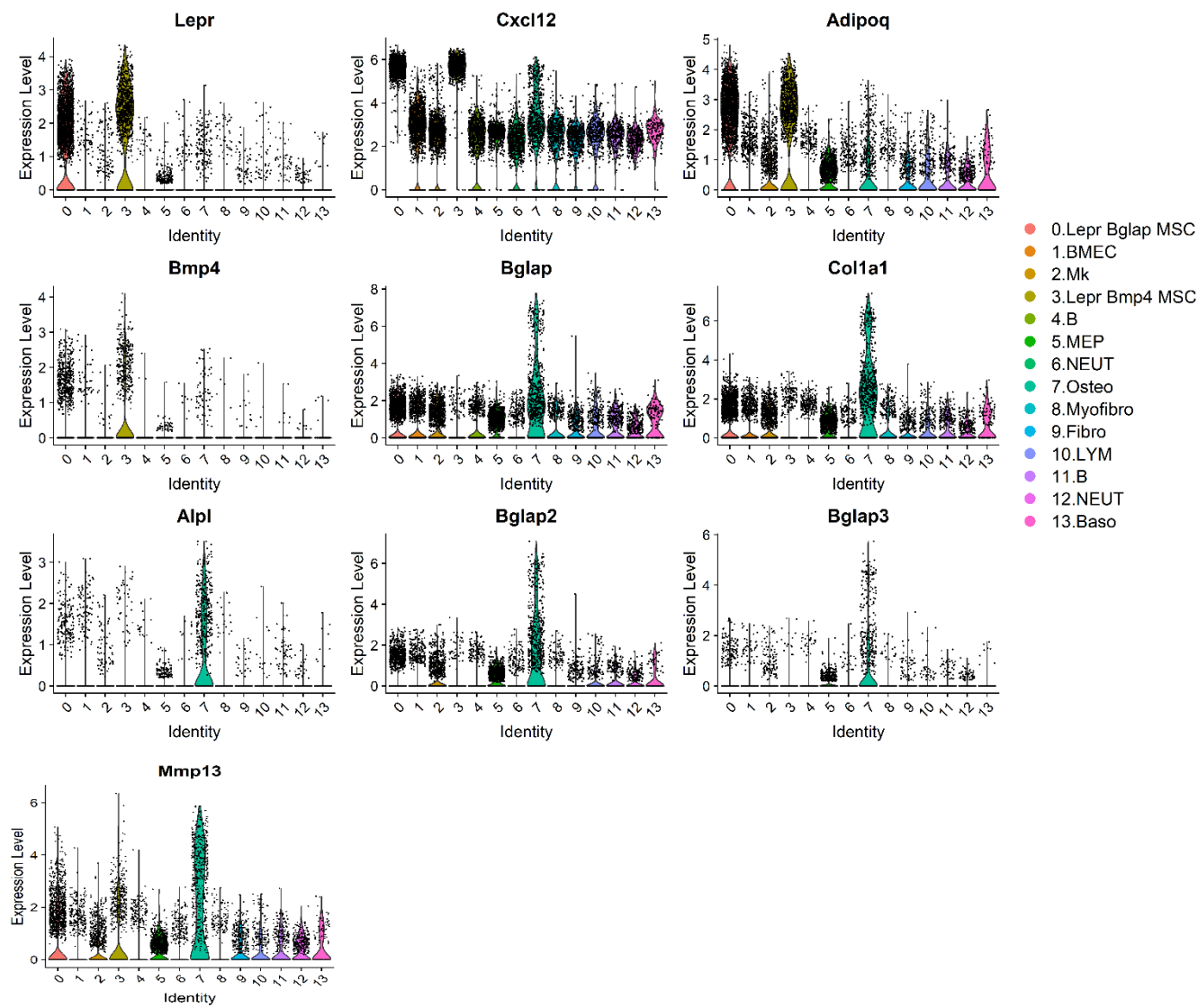

Supplement: Supplementary file 7 — Figure S5 [file 41413_2024_362_MOESM7_ESM.pdf]

Figure S6

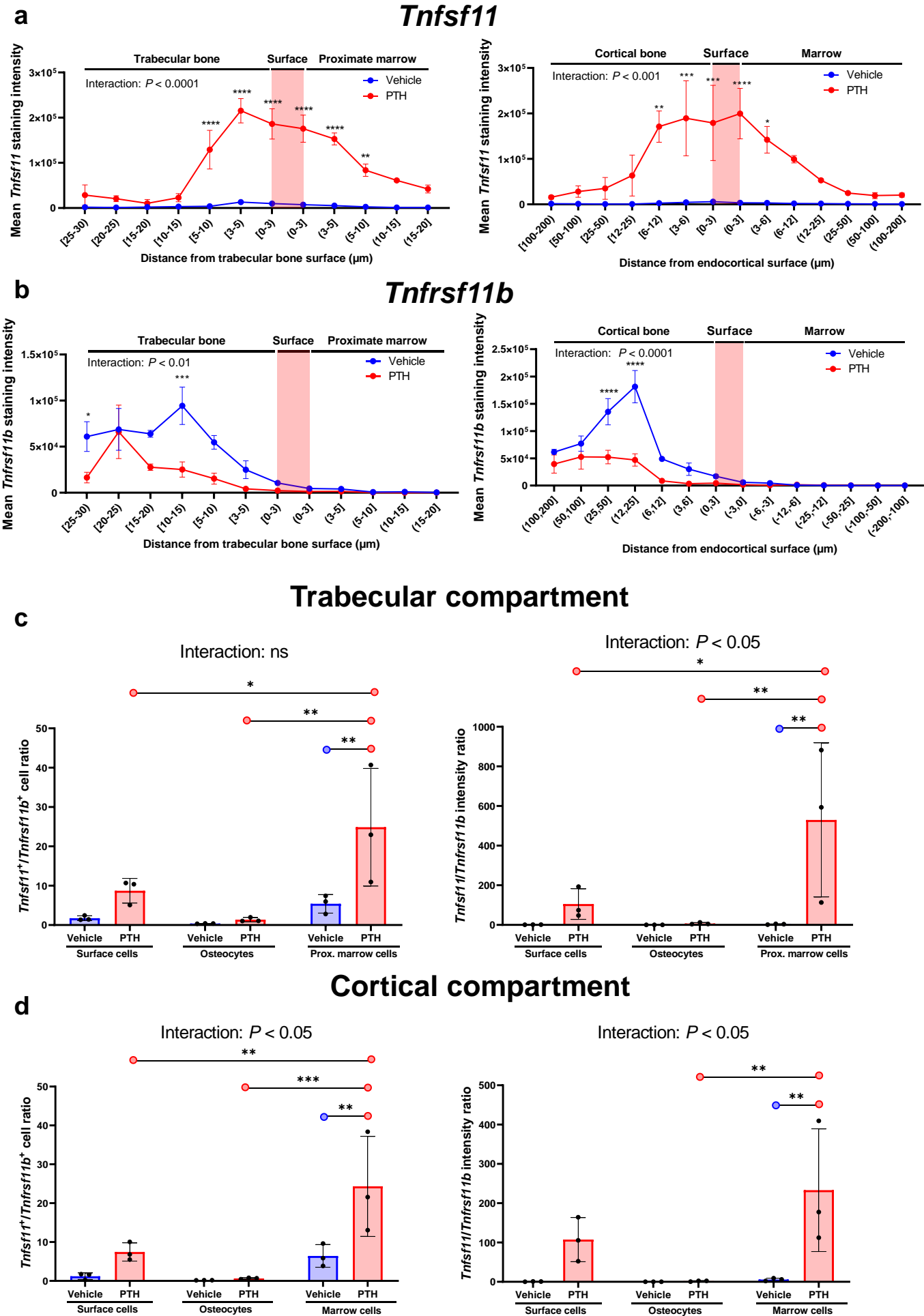

Supplement: Supplementary file 8 — Figure S6 [file 41413_2024_362_MOESM8_ESM.pdf]

Figure S7 – Male vs females RANKL/OPG WT mice

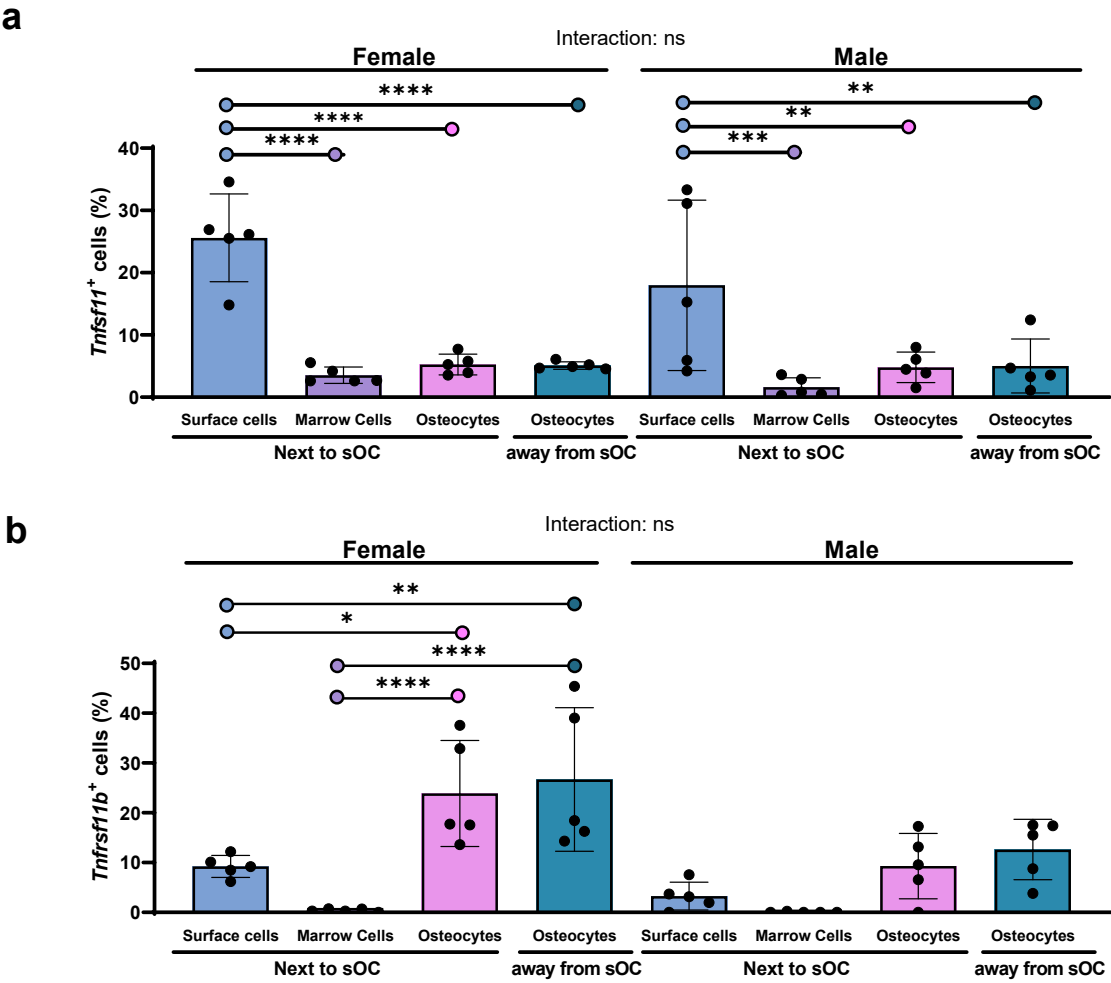

Supplement: Supplementary file 9 — Figure S7 [file 41413_2024_362_MOESM9_ESM.pdf]

Figure S8

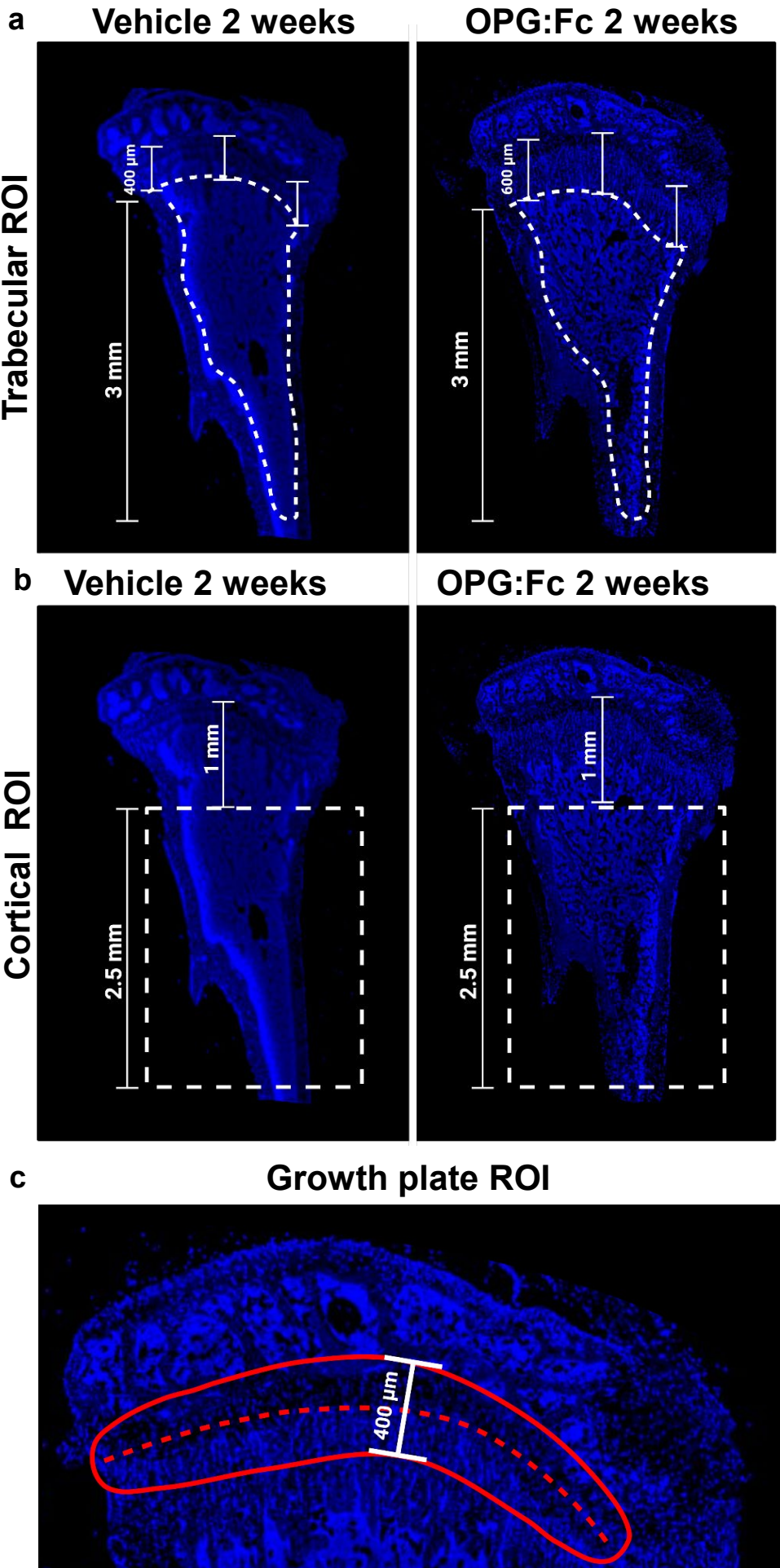

Supplement: Supplementary file 10 — Figure S8 [file 41413_2024_362_MOESM10_ESM.pdf]

Figure S9

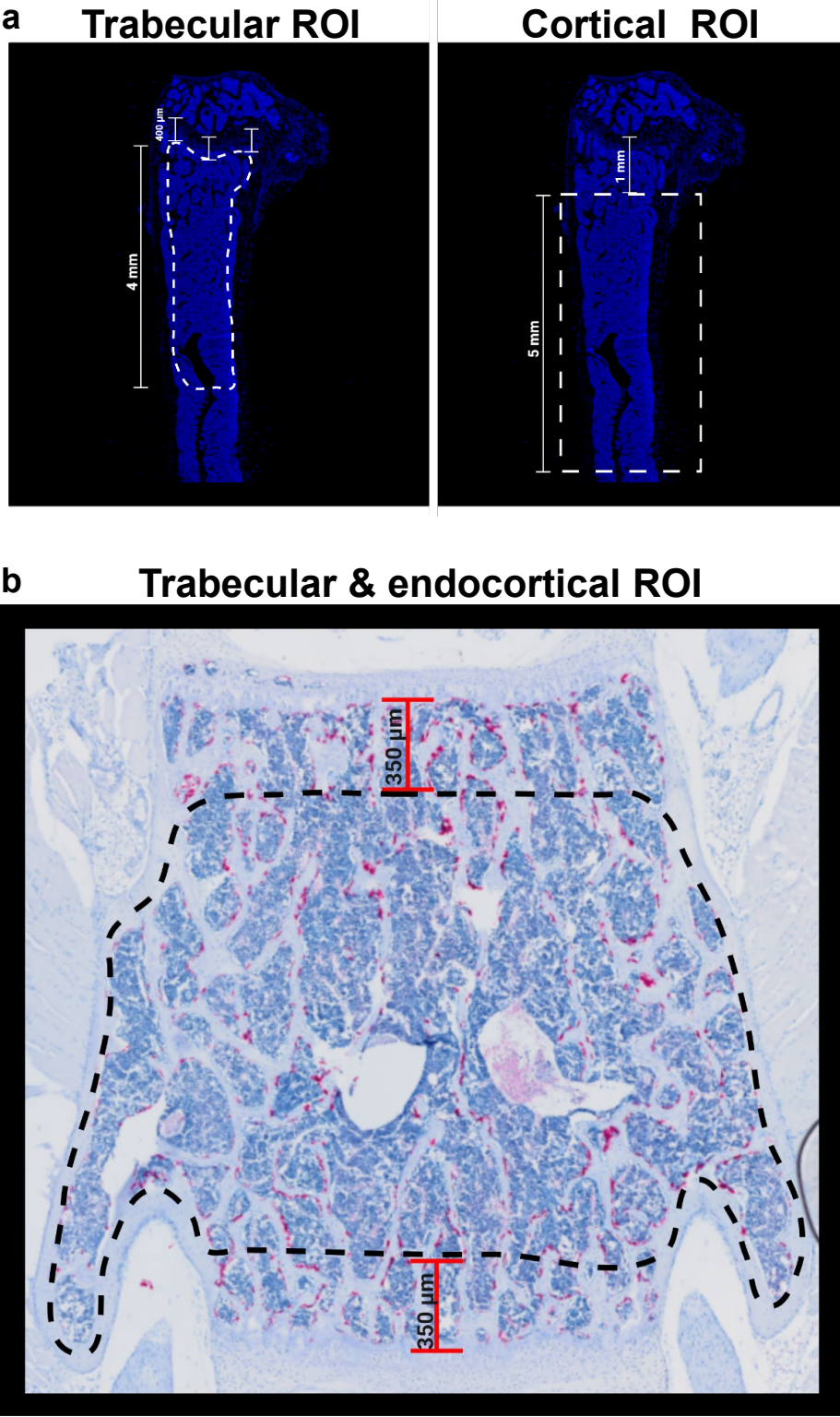

Supplement: Supplementary file 11 — Figure S9 [file 41413_2024_362_MOESM11_ESM.pdf]
